# Supplementary material for: Long-term trajectories of back pain: cohort study with 7-year follow-up
Source: BMJ Open. 2013 Dec 11;3(12):e003838. doi: 10.1136/bmjopen-2013-003838 (PMC3863121; doi:10.1136/bmjopen-2013-003838)
Supplement: Author's manuscript [file bmjopen-2013-003838.draft_revisions.pdf]

Long-term trajectories of back pain: cohort study with seven year follow-up

Journal:	*BMJ Open*
Manuscript ID:	bmjopen-2013-003838
Article Type:	Research
Date Submitted by the Author:	16-Aug-2013
Complete List of Authors:	Dunn, Kate; Keele University, Arthritis Research UK Primary Care Centre<br>Campbell, Paul; Keele University, Arthritis Research UK Primary Care Centre<br>Jordan, Kelvin; Keele University, Arthritis Research UK Primary Care Centre
**Primary Subject Heading**:	Epidemiology
Secondary Subject Heading:	Rheumatology
Keywords:	EPIDEMIOLOGY, PRIMARY CARE, RHEUMATOLOGY

SCHOLARONE™ Manuscripts

For peer review only

Title: Long-term trajectories of back pain: cohort study with seven year follow-up

Authors: Kate M Dunn*, Paul Campbell*, Kelvin P Jordan*

Author affiliations

*Arthritis Research UK Primary Care Centre, Primary Care Sciences, Keele University, Staffordshire, ST5 5BG, UK.

Corresponding Authors Details

Dr Kate M Dunn, Arthritis Research UK Primary Care Centre, Primary Care Sciences, Keele University, Staffordshire, ST5 5BG, UK.

Tel: + 44 (0) 1782 734703; Fax: + 44 (0) 1782 733911, Email: k.m.dunn@keele.ac.uk

http://www.keele.ac.uk/research/pchs/pcmrc/

Keywords: Low Back Pain; Prospective Studies; Cluster Analysis; Longitudinal Studies; Pain Measurement.

Word count: 2560.

For peer review only

Abstract

Objective: To describe long-term trajectories of back pain.

Design: 7-year follow-up of participants in a cohort study.

Setting: Primary care practices in Staffordshire, UK.

Participants: 228 people consulting their GP with back pain, on whom information on 6-month back pain trajectories had been collected during 2001-3, and who had valid consent and contact details in 2009-10, were contacted. 155 participants (68% of those contacted) responded and provided sufficient data for primary analyses.

Outcome measures: Trajectories based on patients' self-reports of back pain, identified using Longitudinal Latent Class Analysis. Trajectories characterised using information on disability, psychological status and presence of other symptoms.

Results: Four clusters with different back pain trajectories at follow-up were identified: (i) no or occasional mild pain, (ii) persistent mild, (iii) fluctuating, and (iv) persistent severe pain. Trajectory clusters differed significantly from each other in terms of disability, psychological status and other symptoms. Most participants remained in a similar trajectory as seven years previously (weighted kappa 0.54; 95% CI 0.42, 0.65).

Conclusions: Most people with back pain follow a particular pain trajectory over long time periods, and do not have frequently recurring or widely fluctuating patterns. Findings raise questions about standard divisions into acute and chronic back pain. A new framework for understanding the course of back pain is proposed.

For peer review only - http://bmjopen.bmj.com/site/about/guidelines.xhtml

Article Summary

Article focus

- Most research studies have limited follow-up in terms of frequency of data collection and long-term timing
- Previous work has used frequent data collection points to identify new short-term trajectories of back pain
- This study aimed to carry out long-term follow-up of people in those trajectories to identify the long-term course and trajectories of back pain.

Key messages

- Four clusters with different back pain trajectories and characteristics at follow-up were identified: (i) no or occasional mild pain, (ii) persistent mild, (iii) fluctuating, and (iv) persistent severe pain.
- Most participants remained in a similar trajectory as seven years previously, indicating that people with back pain follow a particular pain trajectory over long time periods.
- Findings raise questions about standard divisions into acute and chronic back pain based purely on duration of current episode.

Strengths and limitations of this study

- The study benefits from long-term follow-up, prospective design, frequent follow-up during study periods, robust analyses and use of validated questionnaire instruments.
- The study was limited by loss to follow-up, meaning restricted numbers for full analysis, but multiple imputation was used to investigate the implications of this.
- Data collection phases were 7-years apart, and similar information about trajectories in the interim period is unavailable.

For peer review only

Introduction

Back pain is common – it has been recently highlighted as the single leading cause of years lived with disability worldwide (1) and many people experience pain over long periods. Among primary care consulters, 38% report having their symptoms for over three years.(2) Even among people in primary care with acute back pain, 75% report previous back pain,(3) indicating that even if not constantly present, back pain is a long-term experience. This has led to a suggestion to use a longer-term, lifecourse approach to studying back pain.(4)

The long-term experience of back pain is often not addressed by researchers. In a recent review of back pain prognosis, only 1 of the 33 included studies had follow-up beyond a year.(5) Studies with shorter term follow-up can only represent a compressed view of the long-term pain experience. The few longer-term studies have limited numbers of follow-up points,(6,7,8) Knowledge of prognosis is important, as stratifying back pain management based on risk of poor prognosis can be clinically and cost-effective,(9) with benefits for targeting early treatment and referrals. However previous research is unable to fully reflect the detailed course of back pain over time, or inform about long-term prognosis.

In 2001-2 we studied a cohort of people consulting in primary care with back pain.(10) We identified four distinct clusters of people with different trajectories: (i) recovering, (ii) persistent mild, (iii) fluctuating pain, (iv) severe chronic back pain. Duration of back pain at baseline increased with rising severity of trajectory, potentially indicating phases of increasing severity in the long-term course. This is supported by models of stages of back

For peer review only

For peer review only - http://bmjopen.bmj.com/site/about/guidelines.xhtml

pain chronicity (11) and degeneration with age.(12) Alternatively, trajectories could represent distinct groups with stable long-term pain. We aimed to describe long-term trajectories of back pain through the seven year follow-up of a cohort of back pain patients.

Methods

For peer review only

This is a follow-up of participants in a back pain cohort study whose short term (6 month) back pain trajectories had been derived in 2001-2.(10)

Study participants

The original study identified people aged 30-59 years consulting with back pain at one of five general practices in North Staffordshire, UK, during 2001-2. Details are published elsewhere.(13) Briefly, participants returning baseline questionnaires and consenting to follow-up were sent monthly questionnaires. Those returning four or more questionnaires during the first six months were included in a longitudinal latent class analysis to determine trajectories of back pain.(10) Of the 342 participants in this original analysis, 73% (n=250) gave their consent to be contacted again. In 2009, current contact details were not available for 22 (6%), leaving 228 people from the original analysis invited to take part at seven year follow-up.

Data collection at seven years

Self-completion questionnaires were mailed to the 228 study participants (seven year baseline mailing) with reminders at two and four weeks, and brief questionnaires for non-responders at six weeks. Participants giving informed consent were sent brief monthly questionnaires for six months (the same data collection technique as the original study).

All questionnaires contained the same key measures. Pain intensity was measured using the mean of three 0-10 numerical rating scales.(14) Disability was measured using the modified 23-item Roland-Morris Disability Questionnaire.(15) These instruments were used in the original study,(10) and there is evidence of reliability in UK primary care back pain patients.(16) The Chronic Pain Grade classified individuals into grades of chronic pain;(17) this was included in the brief seven year baseline mailing for non-responders. Back pain duration was recalled time since the last pain-free month.(18)

The Hospital Anxiety and Depression Scale was used to assess psychological status.(19) It produces scores from 0-21, with higher scores indicating more severe symptoms. Insomnia was defined as reporting having trouble falling or staying asleep, waking up several times a night, or waking up feeling tired on most nights.(20) This definition has been used previously in pain samples.(21) Somatic symptoms were measured using the 15-item Patient Health Questionnaire (22) which is scored from 0 (not bothered with any symptoms) to 30 (bothered a lot with 15 symptoms). Leg pain was self-reported pain travelling from the back to the

For peer review only - http://bmjopen.bmj.com/site/about/guidelines.xhtml

leg(s), and upper body pain was self-reported pain in the shoulder, arm, neck or head, during the previous two weeks.

Analysis

Two primary analysis groups were formed from responders to this seven-year follow-up study. Group one participants returned the seven year baseline questionnaire plus three or more questionnaires from months one to six. Group two included participants with insufficient seven year follow-up data for full analyses, but who provided adequate information for multiple imputation to be carried out.

For Group one participants, monthly back pain intensity scores were trichotomized into no pain (scoring less than one), mild-moderate pain, and high pain (scored five or more). Longitudinal latent class analysis was used to group participants into clusters based on the trajectory of their back pain over these six months as in the original study.(10) In longitudinal latent class analysis, each participant is allocated to the cluster best matching their pain profile, based on each participant's probability of belonging to each cluster, with participants allocated to the cluster for which they have the largest probability. Participants should be clearly assigned to a single cluster with high probability. Cluster-specific probabilities of having each level of pain for each month, given membership of that cluster, allow development of pain pathways for each cluster. See appendix for more details.

For peer review only - http://bmjopen.bmj.com/site/about/guidelines.xhtml

For peer review only

For Group two participants, the multiple imputation procedure in Stata/IC v11.1 software with 50 imputations, through a multinomial logistic regression, was used to impute membership of the seven year clusters identified for Group one. Information on cluster from the original study, plus outcome measures from the seven year baseline questionnaire were used to impute cluster membership.

Membership of clusters from both study phases (original and seven year follow-up) were compared to investigate long-term patterns of trajectory membership. Stability of cluster membership was assessed using weighted kappa. Kappa can be interpreted as agreement (stability) between original and seven year follow-up cluster memberships beyond chance, with values of 1 indicating perfect agreement and 0 indicating agreement no better than chance. The seven year derived clusters (actual or imputed) were compared on the key measures of the seven year baseline questionnaire, using simple linear or logistic regression as appropriate through the multiple imputation estimate commands in Stata/IC v11.1.

In order to address potential issues from loss to follow-up from the original 2001-3 trajectories analysis, an additional Group three was formed. This included everyone from the original analysis who was not included in the primary analysis at 7 years (above): seven-year responders who provided insufficient data, non-responders at seven years, people who could not be traced, and those not giving consent to follow-up. Groups one and two combined were compared to Group three on baseline demographic, pain, anxiety and depression from the original study using t-tests or chi-squared tests as appropriate.

For peer review only - http://bmjopen.bmj.com/site/about/guidelines.xhtml

As sensitivity analysis, seven year cluster membership was imputed for Group two and Group three participants using information from the original study (baseline Roland-Morris Disability Questionnaire, Chronic Pain Grade, pain duration and original longitudinal latent class analysis cluster). Comparisons between the original cluster and seven year actual or imputed cluster membership for participants across all three groups were performed.

Ethics Statement

The original study and the 2009-10 follow-up phase were independently approved by North Staffordshire Local Research Ethics Committee and South Staffordshire Research Ethics Committee respectively.

Results

Primary analyses were carried out on 155 responders (68% of the 228 contacted): 112 in Group one (full data available) and 43 in Group two (imputation required).

Clusters at seven year follow-up

The optimal number of clusters resulting from longitudinal latent class analysis was four (see appendix). 84% of Group one participants had an average probability of greater than 0.90 of

For peer review only - http://bmjopen.bmj.com/site/about/guidelines.xhtml

being allocated to their assigned cluster, indicating distinct classification. Group two participants were allocated to these clusters using multiple imputation.

The estimated probability of monthly levels of pain within clusters is shown in Table 1. The first cluster (31% of Group one and Group two) mostly had no pain (estimated monthly probabilities of no pain 0.65-0.87), with occasional mild episodes (cluster labelled 'no or occasional pain'). The second cluster (37%) had mild pain intensity throughout, with monthly probabilities of mild pain between 0.69-0.91 ('persistent mild pain'). The third cluster (11%) had pain fluctuating between mild and high levels, ('fluctuating pain'). The final cluster (21%) had high pain intensity levels throughout, with monthly probabilities of high pain between 0.79-0.98 ('persistent severe pain').

Comparison of clusters from original study and seven year follow-up

The identified trajectories of back pain intensity for the original study and the seven year follow-up are illustrated in Figure 1.

Most participants stayed in a similar cluster between the two study phases (weighted kappa 0.54 (95% confidence interval (CI) 0.42, 0.65)) (Table 2). 74% (95% CI 57%, 92%) of those originally in the most severe trajectory remained in an equivalent cluster at seven years. Over half the participants in the two mildest clusters in the original study (recovering: 59%; 95% CI 44%, 74%; persistent mild pain: 56%; 95% CI 40%, 73%) stayed in the most comparable

For peer review only - http://bmjopen.bmj.com/site/about/guidelines.xhtml

trajectory at seven years, and most who changed moved to the other mild trajectory. The fluctuating group in the original study (the smallest group) did not show a stable pattern, with 87% of participants changing cluster, mainly to persistent mild or persistent severe clusters.

Pain intensity, disability and psychological status all differed significantly between the seven year trajectories, with the no or occasional pain cluster having the lowest disability levels and best psychological status, and the persistent severe pain cluster having the highest disability and poorest psychological status (Table 3). Similar statistically significant differences were also present in the original study.(10)

Sensitivity analyses

Group three comprised 25 seven-year responders who provided insufficient data, 48 non-responders at seven years, plus the people from the original study who did not give consent to follow-up (n=92) or could not be traced (n=22). Original study baseline characteristics of the three Groups are shown in Table 4. The only significant difference between participants in Groups one and two and those in Group three was gender, with fewer females in Group three (p=0.04).

Including imputed data from Group three participants as well as Group two made little difference to the estimated relative sizes of the seven year clusters reported above, and gave similar patterns of disability, psychological status and other symptoms.

For peer review only - http://bmjopen.bmj.com/site/about/guidelines.xhtml

Discussion

This study provides unique prospective data on the long-term course of back pain. It suggests that most people remain in a particular pain trajectory, with similar characteristics, when estimated across a seven year period. These findings do not support the hypotheses that there are phases, or degeneration, in the course of back pain over time. Our findings show that widely fluctuating pain is not common (the fluctuating cluster was consistently smallest), and most people have pain patterns varying slightly around their own mean long-term pain. This includes people who recover quickly, and maintain very low (or no) pain, and people who have persistently higher levels of pain. Descriptions of back pain often assume a prevailing pattern of recurrent or fluctuating pain.(23;24) Our findings, and recent qualitative work,(25) provide evidence that these opinions are do not give the full picture. However, our study reports pain trajectories among individuals who have sought healthcare, and although recent work identifying general population trajectories of back pain showed trajectories similar to ours,(26) their fluctuating cluster comprised more of the population (35%).

Strengths of the current study include the long-term nature, prospective design, frequent follow-up during study periods, robust analyses and use of validated questionnaire instruments. However, the study did suffer from loss to follow-up, meaning limited numbers for full analysis. Multiple imputation was used to investigate the implications of this, and participants included in primary analyses were similar to those excluded, but the possibility of selection bias and residual confounding cannot be ruled out. Although this study had frequent follow-up points, data collection phases were 7-years apart, and similar information about trajectories in the interim period is unavailable.

For peer review only - http://bmjopen.bmj.com/site/about/guidelines.xhtml

Few studies have suggested models for long-term change in back pain. Our study gives some support to the model by Raspe et al,(11) as worsening back pain trajectory was significantly associated with more disability, distress, other pains and symptoms, similar to their model of symptom ‘amplification’. However, the prospective nature of our study indicates that this ‘amplification’ is not related to deterioration over time, but describes underlying differences between groups. In addition, it appears that the spread of pain, further complaints and depressive symptoms increases fairly consistently with increasing severity of pain trajectory, rather than occurring in discrete stages, as in the amplification model.(11;27) Our results also do not support models of degeneration with age,(12) as clusters do not differ by age. Our findings suggest a new framework model for the long-term course of back pain, comprising four different types of back pain trajectory, each with characteristic pain patterns, disability levels, psychological status and wider symptoms.

New research is emerging on the treatment of back pain according to prognostic risk groups,(9) but questions have been raised about timing of risk group allocation.(28) Our research highlights potentially stable groups of people with different pain trajectories and characteristics. Comparison of the two study phases showed that no cluster changed mean Roland-Morris Disability Questionnaire score by over 2.5 points (a recommended clinically important change for back pain). This knowledge could improve allocation of treatment according to prognostic risk. However, collecting data over six months to allocate treatment is not clinically plausible, and work is needed to identify pain trajectories concisely and accurately. An important implication of our findings is that classifying back pain simply as acute or chronic is insufficient. This is apparent when standard chronic pain definitions would

For peer review only - http://bmjopen.bmj.com/site/about/guidelines.xhtml

group people with persistent mild symptoms with people who experience constant high levels of pain and other symptoms. Previous work has also highlighted problems defining acute and chronic pain,(25;29) but clinical guidelines are still formulated on this basis.(30;31) Researchers and clinicians should begin to rely less on standard definitions of back pain.

This study raises questions of when, during the life course, trajectory membership is determined. Adolescent trajectories of back pain showed some similar features to the current study (e.g. a cluster with very high probability of pain), whereas other trajectories indicated development of a pain condition.(32) Comparable trajectories were also identified for headache, facial pain and stomach pain in the adolescent cohort,(32) which indicates potential applicability of these findings to other conditions, particularly non-specific symptoms.(33;34)

Conclusions

We have provided unique evidence on the long-term course of back pain, and suggested a new framework for understanding the course of the condition. There is evidence against phases of change in back pain over time. There are some potential limitations of the study, but, if the results apply to a significant proportion of back pain patients, there are important clinical implications. First, a large proportion of those who do report initial pain recover quickly, but among those who do not, our results show that many will remain in the same trajectory over the longer-term. Second, if people in the most severe trajectories could be identified when seeking healthcare, they could be directed to specific targeted treatments. The current study provides substantial new understanding of the long-term course of back pain, and has the potential to have impact in both research and clinical arenas.

For peer review only - http://bmjopen.bmj.com/site/about/guidelines.xhtml

For peer review only

Table 1. Monthly probability of experiencing each level of back pain based on cluster membership at 7-years

	Cluster (trajectory) from 7-year follow-up analysis			
	No / occasional pain	Persistent mild pain	Fluctuating pain	Persistent severe pain
Baseline				
No pain	0·87	0·15	0·01	0·00
Mild-moderate pain	0·13	0·80	0·51	0·21
High pain	0·00	0·05	0·48	0·79
Month 1				
No pain	0·85	0·06	0·00	0·00
Mild-moderate pain	0·15	0·91	0·62	0·17
High pain	0·00	0·04	0·38	0·83
Month 2				
No pain	0·65	0·06	0·00	0·00
Mild-moderate pain	0·35	0·89	0·12	0·11
High pain	0·00	0·05	0·88	0·89
Month 3				
No pain	0·70	0·07	0·01	0·00
Mild-moderate pain	0·30	0·86	0·58	0·17
High pain	0·00	0·07	0·42	0·83
Month 4				
No pain	0·66	0·09	0·00	0·00
Mild-moderate pain	0·34	0·88	0·29	0·18
High pain	0·00	0·03	0·71	0·82
Month 5				
No pain	0·75	0·26	0·19	0·00
Mild-moderate pain	0·25	0·69	0·73	0·02
High pain	0·00	0·05	0·09	0·98
Month 6				
No pain	0·80	0·16	0·02	0·00
Mild-moderate pain	0·20	0·79	0·66	0·11
High pain	0·00	0·05	0·32	0·89

For peer review only

For peer review only - http://bmjopen.bmj.com/site/about/guidelines.xhtml

Table 2. Cluster membership at 7 years stratified by original study cluster (n=155)

Original study cluster	No. in original study cluster	*n* (%)[a] in each cluster (trajectory) at 7 years			
		No or occasional pain	Persistent mild pain	Fluctuating pain	Persistent severe pain
Recovering	57	34 (59%)	18 (32%)	3 (5%)	2 (4%)
Persistent mild	51	12 (23%)	29 (56%)	8 (15%)	2 (5%)
Fluctuating	16	1 (7%)	6 (38%)	2 (13%)	7 (42%)
Severe chronic	31	0 (0%)	4 (12%)	4 (14%)	23 (74%)

[a] estimated following multiple imputation

Weighted kappa = 0.54 (95% CI 0.42, 0.65)

For peer review only - http://bmjopen.bmj.com/site/about/guidelines.xhtml

For peer review only

Table 3. Characteristics of cluster membership at 7-year baseline follow-up, Group 1 and 2 (n=155)

	Cluster (trajectory) from 7-year follow-up analysis				
	No or occasional pain	Persistent mild pain	Fluctuating pain	Persistent severe pain	p-value
% in cluster	31%	37%	11%	21%	
Age	46·3 (43·9, 48·6)	47·7 (45·5, 50·0)	46·3 (42·1, 50·6)	47·0 (43·7, 50·2)	0·85
Female	65% (51, 80)	63% (50, 77)	68% (43, 93)	63% (45, 81)	0·99
Pain intensity	0·8 (0, 1·8)	2·3 (1·8, 2·8)	4·9 (3·6, 6·3)	6·7 (5·8, 7·6)	<0·001
Leg pain	42% (26, 58)	51% (37, 65)	78% (54, 100)	83% (68, 98)	0·009
Upper body pain	52% (36, 68)	71% (58, 84)	88% (71, 100)	93% (84, 100)	0·004
Disability	2·0 (0, 4·1)	4·3 (3·0, 5·6)	8·7 (5·7, 11·7)	12·9 (10·5, 15·3)	<0·001
Anxiety	5·3 (4·1, 6·4)	6·8 (5·6, 8·0)	6·5 (4·4, 8·6)	8·8 (7·3, 10·3)	0·005
Depression	2·8 (1·8, 3·8)	4·9 (3·8, 6·0)	4·3 (2·8, 5·8)	7·4 (5·9, 8·8)	<0·001
PHQ 15	3·9 (2·6, 5·3)	5·0 (3·9, 6·1)	7·4 (4·3, 10·4)	7·7 (5·8, 9·7)	0·006
Insomnia	27% (12, 42)	42% (28, 57)	75% (51, 98)	80% (65, 96)	<0·001

Figures are mean (95% confidence interval) except female, leg pain, upper body pain and insomnia, which are percentage (95% confidence interval). PHQ-15 = Patient Health Questionnaire.

For peer review only

For peer review only - http://bmjopen.bmj.com/site/about/guidelines.xhtml

Table 4. Original study baseline characteristics of study participants

	Full 7-year follow-up (Group 1: n=112)	Limited 7-year follow-up (Group 2: n=43)	Groups 1 & 2: (n=155)	No 7-year follow-up data available (Group 3: n=187)	*p*-value: Groups 1&2 v. Group 3
Gender (female)[†]	72 (64%)	28 (65%)	100 (65%)	100 (53%)	0·04
Age (years)	46·9 (8·3)	47·0 (7·7)	47·0 (8·1)	47·4 (8·2)	0·63
Pain intensity	4·4 (2·7)	4·5 (2·9)	4·4 (2·8)	4·7 (2·5)	0·26
Disability	9·1 (6·8)	10·7 (6·8)	9·5 (6·8)	10·6 (6·4)	0·14
CPG IV[†]	30 (28%)	17 (40%)	47 (31%)	57 (32%)	0·86
Anxiety	8·2 (4.8)	9·1 (4·6)	8·5 (4·8)	8·6 (4·9)	0·82
Depression	6·1 (4·4)	8·4 (4·9)	6·8 (4·6)	7·5 (4·8)	0·15
Duration of pain[†]					
<= 6 months	42 (38%)	14 (33%)	56 (36%)	51 (28%)	0·10
7-35 months	23 (21%)	14 (33%)	37 (24%)	48 (26%)	
>= 3 years	46 (41%)	15 (35%)	61 (40%)	85 (46%)	
Cluster[†]					
Recovering	42 (38%)	15 (35%)	57 (37%)	47 (25%)	0·10
Persistent mild	34 (30%)	17 (40%)	51 (33%)	71 (38%)	
Fluctuating	13 (12%)	3 (7%)	16 (10%)	29 (16%)	
Severe-chronic	23 (21%)	8 (19%)	31 (20%)	40 (21%)	

Figures are mean (standard deviation) except those marked [†] which are numbers (percentage). CPG IV = Chronic Pain Grade IV

For peer review only - http://bmjopen.bmj.com/site/about/guidelines.xhtml

Figure 1. Trajectories of back pain intensity from original study and 7-year follow-up

For peer review only

For peer review only - http://bmjopen.bmj.com/site/about/guidelines.xhtml

Acknowledgements

We thank all the participants and general practices who participated in the original and follow-up phase of this study. We thank Professor Peter Croft for his comments on drafts of the paper.

Competing Interest statement

The authors have declared that no competing interests exist.

Funding statement

The work was supported by the Wellcome Trust grant number 083572. The funding body had no role in study design, data collection and analysis, decision to publish, or preparation of the manuscript.

Author's contributions

Kate Dunn conceived the study. All authors contributed to the design of the study. Paul Campbell and Kate Dunn coordinated the data collection. Kate Dunn and Kelvin Jordan analysed the data. All authors interpreted the data. Kate Dunn drafted the manuscript and all authors contributed to revisions. Kate Dunn had full access to all of the data in the study and takes responsibility for the integrity of the data and the accuracy of the data analysis. All authors approved the final version of the manuscript submitted for publication.

For peer review only - http://bmjopen.bmj.com/site/about/guidelines.xhtml

For peer review only

<u>Checklist</u>

STROBE statement enclosed.

<u>Data sharing statement</u>

The Arthritis Research UK Primary Care Centre has established data sharing arrangements to support joint publications and other research collaborations. Applications for access to anonymised data from our research databases are reviewed by the Centre's Data Custodian and Academic Proposal (DCAP) Committee and a decision regarding access to the data is made subject to the NRES ethical approval first provided for the study and to new analysis being proposed. Further information on our data sharing procedures can be found on the Centre's website (http://www.keele.ac.uk/pchs/publications/datasharingresources/) or by emailing the Centre's data manager (primarycare.datasharing@keele.ac.uk).

For peer review only

References

1. Vos T, Flaxman AD, Naghavi M, Lozano R, Michaud C et al. Years lived with disability (YLDs) for 1160 sequelae of 289 diseases and injuries 1990—2010: a systematic analysis for the Global Burden of Disease Study 2010. The Lancet 2012;**380**: 2163 – 2196.
2. Dunn KM, Croft PR. The importance of symptom duration in determining prognosis. Pain 2006;**121**:126-132.
3. Henschke N, Maher CG, Refshauge KM, Herbert RD, Cumming RG, Bleasel J et al. Prognosis in patients with recent onset low back pain in Australian primary care: inception cohort study. BMJ 2008;**337**:a171.
4. Dunn KM. Extending conceptual frameworks: life course epidemiology for the study of back pain. BMC Musculoskelet Disord 2010;**11**:23.
5. Costa LD, Maher CG, Hancock MJ, McAuley JH, Herbert RD, Costa LO. The prognosis of acute and persistent low-back pain: a meta-analysis. Canadian Medical Association Journal 2012;**84**:E613-E624.
6. Enthoven P, Skargren E, Carstensen J, Oberg B. Predictive factors for 1-year and 5-year outcome for disability in a working population of patients with low back pain treated in primary care. Pain 2006;**122**:137-144.
7. Burton AK, McClune TD, Clarke RD, Main CJ. Long-term follow-up of patients with low back pain attending for manipulative care-outcomes and predictors. Man Ther 2004;**9**:30-35.
8. Von Korff M, Miglioretti DL. A prognostic approach to defining chronic pain. Pain 2005;**117**:304-313.
9. Hill JC, Whitehurst DG, Lewis M, Bryan S, Dunn KM, Foster NE et al. Comparison of stratified primary care management for low back pain with current best practice (STarT Back): a randomised controlled trial. The Lancet 2011;**378**: 1560-1571.
10. Dunn KM, Jordan K, Croft PR. Characterising the course of low back pain: a latent class analysis. Am J Epidemiol 2006;**163**:754-761.
11. Raspe H, Huppe A, Matthis C. Theories and models of chronicity: on the way to a broader definition of chronic back pain. Schmerz 2003;**17**:359-366.
12. Laurens Rowe M. Backache at work. Fairport, New York: Perinton Press; 1983.
13. Dunn KM, Croft PR. Classification of low back pain in primary care: using "bothersomeness" to identify the most severe cases. Spine 2005;**30**:1887-1892.
14. Dunn KM, Jordan KP, Croft PR. Recall of medication use, self-care activities and pain intensity: a comparison of daily diaries and self-report questionnaires among low back pain patients. Primary Health Care Research & Development 2010;**11**:93-102.
15. Patrick DL, Deyo RA, Atlas SJ, Singer DE, Chapin AM, Keller RB. Assessing health-related quality of life in patients with sciatica. Spine 1995;**20**:1899-1908.

For peer review only

16. Dunn KM, Jordan K, Croft PR. Does questionnaire structure influence response in postal surveys? J Clin Epidemiol 2003;**56**:10-16.

17. Von Korff M, Ormel J, Keefe FJ, Dworkin SF. Grading the severity of chronic pain. Pain 1992;**50**:133-149.

18. Dunn KM, de Vet HCW, Hooper H, Ong BN, Croft PR. Measurement of back pain episode inception in questionnaires: a study combining quantitative and qualitative methods. Journal of Musculoskeletal Pain 2006;**14**:29-37.

19. Zigmond AS, Snaith RP. The Hospital Anxiety and Depression Scale. Acta Psychiatr Scand 1983;**67**:361-370.

20. Jenkins CD, Stanton BA, Niemcryk SJ, Rose RM. A scale for the estimation of sleep problems in clinical research. J Clin Epidemiol 1988;**41**:313-321.

21. Morphy H, Dunn KM, Lewis M, Boardman HF, Croft PR. Epidemiology of insomnia: a longitudinal study in a UK population. Sleep 2007;**30**:274-280.

22. Kroenke K, Spitzer RL, Williams JB. The PHQ-15: validity of a new measure for evaluating the severity of somatic symptoms. Psychosom Med 2002;**64**:258-266.

23. Waddell G. The Back Pain Revolution. Edinburgh: Churchill Livingstone; 1998.

24. Hestbaek L, Leboeuf-Yde C, Manniche C. Low back pain: what is the long-term course? A review of studies of general patient populations. Eur Spine J 2003;**12**:149-165.

25. Young AE, Wasiak R, Phillips L, Gross DP. Workers' perspectives on low back pain recurrence: "It comes and goes and comes and goes, but it's always there". Pain 2011;**152**:204-211.

26. Tamcan O, Mannion AF, Eisenring C, Horisberger B, Elfering A, Müller U. The course of chronic and recurrent low back pain in the general population. Pain 2010;**150**:451-457.

27. Raspe H. (2010) Measuring the impact of chronic pain on populations: a narrative review. In: Croft P, Blyth FM, van der Windt D, editors. Chronic pain epidemiology: From aetiology to public health. 1st ed. Oxford: Oxford University Press. pp. 61-70.

28. Hill JC, Vohora K, Dunn KM, Main CJ, Hay E. Comparing the STarT Back screening tool's subgroup allocation of individual patients with that of independent clinical experts. Clin J Pain 2010;**26**: 783-787.

29. Von Korff M, Dunn KM. Chronic pain reconsidered. Pain 2008;**138**:267-276.

30. Savigny P, Kuntze S, Watson P, Underwood M, Ritchie G, Cotterell M. (2009) Early management of persistent non-specific low back pain. London, National Collaborating Centre for Primary Care and Royal College of General Practitioners.

31. Chou R, Qaseem A, Snow V, Casey D, Cross JT, Jr., Shekelle P et al. Diagnosis and treatment of low back pain: a joint clinical practice guideline from the American College of Physicians and the American Pain Society. Ann Intern Med 2007;**147**:478-491.

32. Dunn KM, Jordan KP, Mancl L, Drangsholt MT, Le Resche L. Trajectories of pain in adolescents: a prospective cohort study. Pain 2011;**152**:66-73.

For peer review only - http://bmjopen.bmj.com/site/about/guidelines.xhtml

33. Aggarwal VR, McBeth J, Zakrzewska JM, Lunt M, Macfarlane GJ. The epidemiology of chronic syndromes that are frequently unexplained: do they have common associated factors? Int J Epidemiol 2006;**35**:468-476.

34. Nimnuan C, Rabe-Hesketh S, Wessely S, Hotopf M. How many functional somatic syndromes? J Psychosom Res 2001;**51**:549-557.

For peer review only

For peer review only - http://bmjopen.bmj.com/site/about/guidelines.xhtml

For peer review only

For peer review only - http://bmjopen.bmj.com/site/about/guidelines.xhtml

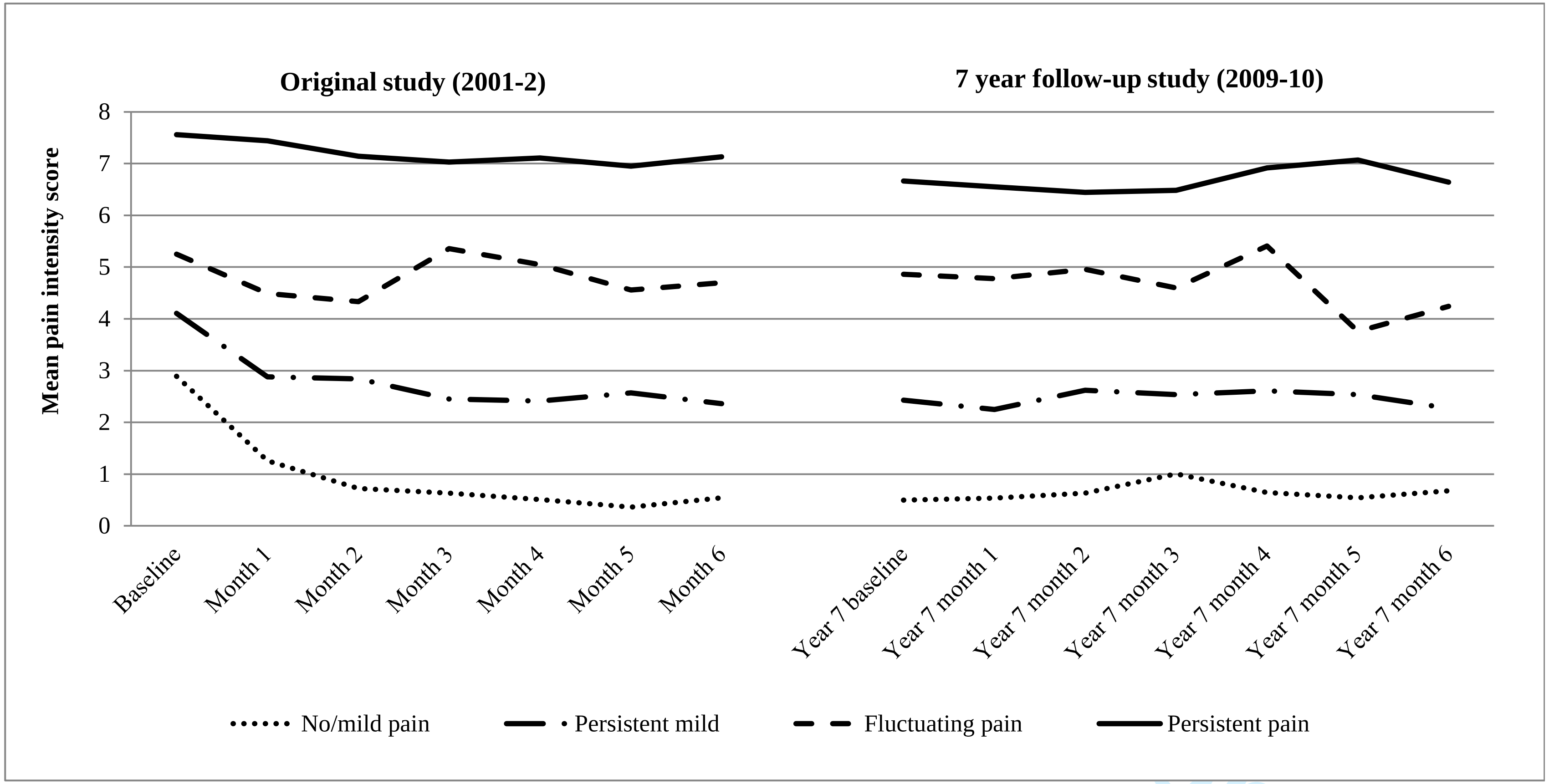
Original study (2001-2)
7 year follow-up study (2009-10)
Mean pain intensity score
8
7
6
5
4
3
2
1
0
Baseline
Month 1
Month 2
Month 3
Month 4
Month 5
Month 6
Year 7 baseline
Year 7 month 1
Year 7 month 2
Year 7 month 3
Year 7 month 4
Year 7 month 5
Year 7 month 6
No/mild pain
Persistent mild
Fluctuating pain
Persistent pain

STROBE 2007 (v4) Statement—Checklist of items that should be included in reports of *cohort studies*

Section/Topic	Item #	Recommendation	Reported on page #
**Title and abstract**	1	(*a*) Indicate the study's design with a commonly used term in the title or the abstract	1
		(*b*) Provide in the abstract an informative and balanced summary of what was done and what was found	2
**Introduction**			
Background/rationale	2	Explain the scientific background and rationale for the investigation being reported	4
Objectives	3	State specific objectives, including any prespecified hypotheses	5
**Methods**			
Study design	4	Present key elements of study design early in the paper	5-7
Setting	5	Describe the setting, locations, and relevant dates, including periods of recruitment, exposure, follow-up, and data collection	5-6
Participants	6	(*a*) Give the eligibility criteria, and the sources and methods of selection of participants. Describe methods of follow-up	5-7
		(*b*) For matched studies, give matching criteria and number of exposed and unexposed	n/a
Variables	7	Clearly define all outcomes, exposures, predictors, potential confounders, and effect modifiers. Give diagnostic criteria, if applicable	6-7
Data sources/ measurement	8*	For each variable of interest, give sources of data and details of methods of assessment (measurement). Describe comparability of assessment methods if there is more than one group	*6-7*
Bias	9	Describe any efforts to address potential sources of bias	7-9
Study size	10	Explain how the study size was arrived at	5-6
Quantitative variables	11	Explain how quantitative variables were handled in the analyses. If applicable, describe which groupings were chosen and why	7-9
Statistical methods	12	(*a*) Describe all statistical methods, including those used to control for confounding	7-9
		(*b*) Describe any methods used to examine subgroups and interactions	7-9
		(*c*) Explain how missing data were addressed	7-9
		(*d*) If applicable, explain how loss to follow-up was addressed	7-9
		(*e*) Describe any sensitivity analyses	9
**Results**			

For peer review only

Participants	13*	(a) Report numbers of individuals at each stage of study—eg numbers potentially eligible, examined for eligibility, confirmed eligible, included in the study, completing follow-up, and analysed	9
		(b) Give reasons for non-participation at each stage	5-6 & 9 & 11
		(c) Consider use of a flow diagram	
Descriptive data	14*	(a) Give characteristics of study participants (eg demographic, clinical, social) and information on exposures and potential confounders	17-18
		(b) Indicate number of participants with missing data for each variable of interest	5-6 & 9 & 11
		(c) Summarise follow-up time (eg, average and total amount)	5-6
Outcome data	15*	Report numbers of outcome events or summary measures over time	9-10
Main results	16	(*a*) Give unadjusted estimates and, if applicable, confounder-adjusted estimates and their precision (eg, 95% confidence interval). Make clear which confounders were adjusted for and why they were included	n/a
		(*b*) Report category boundaries when continuous variables were categorized	6-7
		(*c*) If relevant, consider translating estimates of relative risk into absolute risk for a meaningful time period	n/a
Other analyses	17	Report other analyses done—eg analyses of subgroups and interactions, and sensitivity analyses	11
**Discussion**			
Key results	18	Summarise key results with reference to study objectives	12
**Limitations**			
Interpretation	20	Give a cautious overall interpretation of results considering objectives, limitations, multiplicity of analyses, results from similar studies, and other relevant evidence	12-14
Generalisability	21	Discuss the generalisability (external validity) of the study results	12
**Other information**			
Funding	22	Give the source of funding and the role of the funders for the present study and, if applicable, for the original study on which the present article is based	20

*Give information separately for cases and controls in case-control studies and, if applicable, for exposed and unexposed groups in cohort and cross-sectional studies.

Note: An Explanation and Elaboration article discusses each checklist item and gives methodological background and published examples of transparent reporting. The STROBE checklist is best used in conjunction with this article (freely available on the Web sites of PLoS Medicine at http://www.plosmedicine.org/, Annals of Internal Medicine at http://www.annals.org/, and Epidemiology at http://www.epidem.com/). Information on the STROBE Initiative is available at www.strobe-statement.org.

For peer review only

Long-term trajectories of back pain: cohort study with seven year follow-up

Journal:	*BMJ Open*
Manuscript ID:	bmjopen-2013-003838.R1
Article Type:	Research
Date Submitted by the Author:	11-Oct-2013

Complete List of Authors:	Dunn, Kate; Keele University, Arthritis Research UK Primary Care Centre<br>Campbell, Paul; Keele University, Arthritis Research UK Primary Care Centre<br>Jordan, Kelvin; Keele University, Arthritis Research UK Primary Care Centre

**Primary Subject Heading**:	Epidemiology
Secondary Subject Heading:	Rheumatology
Keywords:	EPIDEMIOLOGY, PRIMARY CARE, RHEUMATOLOGY

SCHOLARONE™
Manuscripts

Title: **Long-term trajectories of back pain: cohort study with seven year follow-up**

Authors: Kate M Dunn*, Paul Campbell*, Kelvin P Jordan*

Author affiliations

*Arthritis Research UK Primary Care Centre, Primary Care Sciences, Keele University, Staffordshire, ST5 5BG, UK.

Corresponding Authors Details

Dr Kate M Dunn, Arthritis Research UK Primary Care Centre, Primary Care Sciences, Keele University, Staffordshire, ST5 5BG, UK.

Tel: + 44 (0) 1782 734703; Fax: + 44 (0) 1782 733911, Email: k.m.dunn@keele.ac.uk

http://www.keele.ac.uk/research/pchs/pcmrc/

Keywords: Low Back Pain; Prospective Studies; Cluster Analysis; Longitudinal Studies; Pain Measurement.

Word count: 2767.

For peer review only - http://bmjopen.bmj.com/site/about/guidelines.xhtml

Abstract

Objective: To describe long-term trajectories of back pain.

Design: Monthly data collection for 6-months at 7-year follow-up of participants in a prospective cohort study.

Setting: Primary care practices in Staffordshire, UK.

Participants: 228 people consulting their GP with back pain, on whom information on 6-month back pain trajectories had been collected during 2001-3, and who had valid consent and contact details in 2009-10, were contacted. 155 participants (68% of those contacted) responded and provided sufficient data for primary analyses.

Outcome measures: Trajectories based on patients' self-reports of back pain, identified using Longitudinal Latent Class Analysis. Trajectories characterised using information on disability, psychological status and presence of other symptoms.

Results: Four clusters with different back pain trajectories at follow-up were identified: (i) no or occasional pain, (ii) persistent mild pain, (iii) fluctuating pain, and (iv) persistent severe pain. Trajectory clusters differed significantly from each other in terms of disability, psychological status and other symptoms. Most participants remained in a similar trajectory as seven years previously (weighted kappa 0.54; 95% CI 0.42, 0.65).

Conclusions: Most people with back pain appear to follow a particular pain trajectory over long time periods, and do not have frequently recurring or widely fluctuating patterns. Results are limited by lack of information about the time between data collection periods, and by loss to follow-up. However, findings do raise questions about standard divisions into acute and chronic back pain. A new framework for understanding the course of back pain is proposed.

For peer review only

For peer review only - http://bmjopen.bmj.com/site/about/guidelines.xhtml

Article Summary

Article focus

- Most research studies have limited follow-up in terms of frequency of data collection and long-term timing
- Previous work has used frequent data collection points to identify new short-term trajectories of back pain
- This study aimed to carry out long-term follow-up of people in those trajectories to identify the long-term course and trajectories of back pain.

Key messages

- Four clusters with different back pain trajectories and characteristics at follow-up were identified: (i) no or occasional pain, (ii) persistent mild pain, (iii) fluctuating pain, and (iv) persistent severe pain.
- Most participants remained in a similar trajectory as seven years previously, indicating that people with back pain follow a particular pain trajectory over long time periods.
- Findings raise questions about standard divisions into acute and chronic back pain based purely on duration of current episode.

Strengths and limitations of this study

- The study benefits from long-term follow-up, prospective design, frequent follow-up during study periods, robust analyses and use of validated questionnaire instruments.
- The study was limited by loss to follow-up, meaning restricted numbers for full analysis, but multiple imputation was used to investigate the implications of this.
- Data collection phases were 7-years apart, and similar information about trajectories in the interim period is unavailable.

For peer review only

Introduction

Back pain is common – it has been recently highlighted as the single leading cause of years lived with disability worldwide (1) and many people experience pain over long periods. Among primary care consulters, 38% report having their symptoms for over three years.(2) Even among people in primary care with acute back pain, 75% report previous back pain,(3) indicating that even if not constantly present, back pain is a long-term experience. This has led to a suggestion to use a longer-term, lifecourse approach to studying back pain.(4)

The long-term experience of back pain is often not addressed by researchers. In a recent review of back pain prognosis, only 1 of the 33 included studies had follow-up beyond a year.(5) Studies with shorter term follow-up can only represent a compressed view of the long-term pain experience. The few longer-term studies have limited numbers of follow-up points,(6,7,8) Knowledge of prognosis is important, as stratifying back pain management based on risk of poor prognosis can be clinically and cost-effective,(9) with benefits for targeting early treatment and referrals. However previous research is unable to fully reflect the detailed course of back pain over time, or inform about long-term prognosis.

In 2001-2 we studied a cohort of people consulting in primary care with back pain.(10) We identified four distinct clusters of people with different trajectories: (i) recovering, (ii) persistent mild, (iii) fluctuating pain, (iv) severe chronic back pain. Duration of back pain at baseline increased with rising severity of trajectory, potentially indicating phases of increasing severity in the long-term course. This is supported by models of stages of back

For peer review only

For peer review only - http://bmjopen.bmj.com/site/about/guidelines.xhtml

pain chronicity (11) and degeneration with age.(12) Alternatively, trajectories could represent distinct groups with stable long-term pain. We aimed to describe long-term trajectories of back pain through a 6 month follow-up period of a cohort of back pain patients previously studied seven years earlier.

Methods

This is a follow-up of participants in a back pain cohort study whose short term (6 month) back pain trajectories had been derived in 2001-2.(10)

Study participants

The original study identified people aged 30-59 years consulting with back pain at one of five general practices in North Staffordshire, UK, during 2001-2. Details are published elsewhere.(13) Briefly, participants returning baseline questionnaires and consenting to follow-up were sent monthly questionnaires. Those returning four or more questionnaires during the first six months were included in a longitudinal latent class analysis to determine trajectories of back pain.(10) Of the 342 participants in this original analysis, 73% (n=250) gave their consent to be contacted again. In 2009, current contact details were not available for 22 (6%), leaving 228 people from the original analysis invited to take part at seven year follow-up.

For peer review only - http://bmjopen.bmj.com/site/about/guidelines.xhtml
For peer review only

Data collection at seven years

Self-completion questionnaires were mailed to the 228 study participants (seven year baseline mailing) with reminders at two and four weeks, and brief questionnaires for non-responders at six weeks. Participants giving informed consent were sent brief monthly questionnaires for six months (the same data collection technique as the original study).

All questionnaires contained the same key measures. Pain intensity was measured using the mean of three 0-10 numerical rating scales.(14) Disability was measured using the modified 23-item Roland-Morris Disability Questionnaire (RMDQ).(15) These instruments were used in the original study,(10) and there is evidence of reliability in UK primary care back pain patients.(16) The Chronic Pain Grade classified individuals into grades of chronic pain;(17) this was included in the brief seven year baseline mailing for non-responders. Back pain duration was recalled time since the last pain-free month.(18)

The Hospital Anxiety and Depression Scale (HADS) was used to assess psychological status.(19) It produces scores from 0-21, with higher scores indicating more severe symptoms. Insomnia was defined as reporting having trouble falling or staying asleep, waking up several times a night, or waking up feeling tired on most nights.(20) This definition has been used previously in pain samples.(21) Somatic symptoms were measured using the 15-item Patient Health Questionnaire (PHQ-15) (22) which is scored from 0 (not bothered with any symptoms) to 30 (bothered a lot with 15 symptoms). Leg pain was self-

For peer review only - http://bmjopen.bmj.com/site/about/guidelines.xhtml

For peer review only

reported pain travelling from the back to the leg(s), and upper body pain was self-reported pain in the shoulder, arm, neck or head, during the previous two weeks.

Analysis

Two primary analysis groups were formed from responders to this seven-year follow-up study. Group one participants returned the seven year baseline questionnaire plus three or more questionnaires from months one to six. Group two included participants with insufficient seven year follow-up data for full analyses, but who provided adequate information for multiple imputation to be carried out.

For Group one participants, monthly back pain intensity scores were trichotomized into no pain (scoring less than one), mild-moderate pain, and high pain (scored five or more). Longitudinal latent class analysis was used to group participants into clusters based on the trajectory of their back pain over these six months as in the original study.(10) In longitudinal latent class analysis, each participant is allocated to the cluster best matching their pain profile, based on each participant's probability of belonging to each cluster, with participants allocated to the cluster for which they have the largest probability. Participants should be clearly assigned to a single cluster with high probability. Cluster-specific probabilities of having each level of pain for each month, given membership of that cluster, allow development of pain pathways for each cluster. See appendix for more details.

For peer review only - http://bmjopen.bmj.com/site/about/guidelines.xhtml

For Group two participants, the multiple imputation procedure in Stata/IC v11.1 software with 50 imputations, through a multinomial logistic regression, was used to impute membership of the seven year clusters identified for Group one. Information on cluster from the original study, plus outcome measures from the seven year baseline questionnaire were used to impute cluster membership.

Membership of clusters from both study phases (original and seven year follow-up) were compared to investigate long-term patterns of trajectory membership. Stability of cluster membership was assessed using weighted kappa. Kappa can be interpreted as agreement (stability) between original and seven year follow-up cluster memberships beyond chance, with values of 1 indicating perfect agreement and 0 indicating agreement no better than chance. The seven year derived clusters (actual or imputed) were compared on the key measures of the seven year baseline questionnaire, using simple linear or logistic regression as appropriate through the multiple imputation estimate commands in Stata/IC v11.1.

In order to address potential issues from loss to follow-up from the original 2001-3 trajectories analysis, an additional Group three was formed. This included everyone from the original analysis who was not included in the primary analysis at 7 years (above): seven-year responders who provided insufficient data, non-responders at seven years, people who could not be traced, and those not giving consent to follow-up. Groups one and two combined were compared to Group three on baseline demographic, pain, anxiety and depression from the original study using t-tests or chi-squared tests as appropriate.

For peer review only - http://bmjopen.bmj.com/site/about/guidelines.xhtml

As sensitivity analysis, seven year cluster membership was imputed for Group two and Group three participants using information from the original study (baseline Roland-Morris Disability Questionnaire, Chronic Pain Grade, pain duration and original longitudinal latent class analysis cluster). Comparisons between the original cluster and seven year actual or imputed cluster membership for participants across all three groups were performed.

Ethics Statement

The original study and the 2009-10 follow-up phase were independently approved by North Staffordshire Local Research Ethics Committee and South Staffordshire Research Ethics Committee respectively.

Results

Primary analyses were carried out on 155 responders (68% of the 228 contacted): 112 in Group one (full data available) and 43 in Group two (imputation required).

Clusters at seven year follow-up

The optimal number of clusters resulting from longitudinal latent class analysis was four (see appendix). 84% of Group one participants had an average probability of greater than 0.90 of

For peer review only - http://bmjopen.bmj.com/site/about/guidelines.xhtml

being allocated to their assigned cluster, indicating distinct classification. Group two participants were allocated to these clusters using multiple imputation.

The estimated probability of monthly levels of pain within clusters is shown in Table 1. These monthly probabilities of pain can be interpreted to describe the occurrence of pain, for example, a probability of mild-moderate pain of 0.13 at baseline for the first Cluster indicates that one in every eight people in that group are likely to have experienced mild-moderate pain that month. The first cluster identified (31% of Group one and Group two) mostly had no pain (estimated monthly probabilities of no pain 0.65-0.87), with occasional mild episodes (cluster labelled 'no or occasional pain'). Participants in this cluster generally reported no pain on at least 4 occasions over the six months and did not report high pain. The second cluster (37%) had mild pain intensity most of the time, with a maximum of 1-2 months of no pain; only 17% of the cluster ever reported high pain. Their monthly probabilities of mild pain were between 0.69-0.91 ('persistent mild pain'). The third cluster (11%) had pain fluctuating between mild and high levels ('fluctuating pain'), and rarely reported no pain. The final cluster (21%) had high pain intensity levels throughout, with monthly probabilities of high pain between 0.79-0.98 ('persistent severe pain'), and never reported no pain.

Comparison of clusters from original study and seven year follow-up

The identified trajectories of back pain intensity for the original study and the seven year follow-up are illustrated in Figure 1.

For peer review only - http://bmjopen.bmj.com/site/about/guidelines.xhtml

Most participants stayed in a similar cluster between the two study phases (weighted kappa 0.54 (95% confidence interval (CI) 0.42, 0.65)) (Table 2). 74% (95% CI 57%, 92%) of those originally in the most severe trajectory remained in an equivalent cluster at seven years. Over half the participants in the two mildest clusters in the original study (recovering: 59%; 95% CI 44%, 74%; persistent mild pain: 56%; 95% CI 40%, 73%) stayed in the most comparable trajectory at seven years, and most who changed moved to the other mild trajectory. The fluctuating group in the original study (the smallest group) did not show a stable pattern, with 87% of participants changing cluster, mainly to persistent mild or persistent severe clusters.

Pain intensity, disability and psychological status all differed significantly between the seven year trajectories, with the no or occasional pain cluster having the lowest disability levels (mean RMDQ score 2.0), least pain intensity (mean 0.8) and best psychological status (mean HADS depression score 2.8), and the persistent severe pain cluster having the highest disability (mean RMDQ score 12.9), worst pain intensity (mean 6.7) and poorest psychological status (mean HADS depression score 7.4) (Table 3). Similar statistically significant differences were also present in the original study.(10) The clusters also differed significantly in terms of the presence of somatic symptoms and insomnia, with the mean symptom score (PHQ-15) ranging from 3.9 in the no or occasional pain group to 7.7 in the persistent severe pain cluster, and the proportion classified with insomnia ranging from 27% to 80%.

Sensitivity analyses

For peer review only - http://bmjopen.bmj.com/site/about/guidelines.xhtml

Group three comprised 25 seven-year responders who provided insufficient data, 48 non-responders at seven years, plus the people from the original study who did not give consent to follow-up (n=92) or could not be traced (n=22). Original study baseline characteristics of the three Groups are shown in Table 4. The only significant difference between participants in Groups one and two and those in Group three was gender, with fewer females in Group three (p=0.04).

Including imputed data from Group three participants as well as Group two made little difference to the estimated relative sizes of the seven year clusters reported above, and gave similar patterns of disability, psychological status and other symptoms.

For peer review only

Discussion

This study provides unique prospective data on the long-term course of back pain. It suggests that most people remain in a particular pain trajectory, with similar characteristics, when estimated across a seven year period. These findings do not support the hypotheses that there are phases, or degeneration, in the course of back pain over time. Our findings show that widely fluctuating pain is not common (the fluctuating cluster was consistently smallest), and most people have pain patterns varying slightly around their own mean long-term pain. This includes people who recover quickly, and maintain very low (or no) pain, and people who have persistently higher levels of pain. Descriptions of back pain often assume a prevailing pattern of recurrent or fluctuating pain.(23;24) Our findings, and recent qualitative work,(25) provide evidence that these opinions do not give the full picture. However, our study reports pain trajectories among individuals who have sought healthcare, and although recent work identifying general population trajectories of back pain showed trajectories similar to ours,(26) their fluctuating cluster comprised more of the population (35%).

Strengths of the current study include the long-term nature, prospective design, frequent follow-up during study periods, robust analyses and use of validated questionnaire instruments. However, the study did suffer from loss to follow-up, meaning limited numbers for full analysis. Multiple imputation was used to investigate the implications of this, and participants included in primary analyses were similar to those excluded, but the possibility of selection bias and residual confounding cannot be ruled out. Although this study had frequent follow-up points, data collection phases were 7-years apart, and similar information about trajectories in the interim period is unavailable.

For peer review only - http://bmjopen.bmj.com/site/about/guidelines.xhtml

Few studies have suggested models for long-term change in back pain. Our study gives some support to the model by Raspe et al,(11) as worsening back pain trajectory was significantly associated with more disability, distress, other pains and symptoms, similar to their model of symptom ‘amplification’. However, the prospective nature of our study indicates that this ‘amplification’ is not related to deterioration over time or stages of change, but describes underlying differences between groups of people whose general pattern of pain does not appear to change over time. In addition, it appears that the spread of pain, further complaints and depressive symptoms increases fairly consistently with increasing severity of pain trajectory, rather than occurring in discrete stages, as in the amplification model.(11;27) Our results also do not support models of degeneration with age,(12) as clusters do not differ by age. Our findings suggest a new framework model for the long-term course of back pain, comprising four different types of back pain trajectory, each with characteristic pain patterns, disability levels, psychological status and wider symptoms.

New research is emerging on the treatment of back pain according to prognostic risk groups,(9) but questions have been raised about timing of risk group allocation.(28) Our research highlights potentially stable groups of people with different pain trajectories and characteristics. Comparison of the two study phases showed that no cluster changed mean Roland-Morris Disability Questionnaire score by over 2.5 points (a recommended clinically important change for back pain). This knowledge could improve allocation of treatment according to prognostic risk. However, collecting data over six months to allocate treatment is not clinically plausible, and work is needed to identify pain trajectories concisely and accurately. An important implication of our findings is that classifying back pain simply as

For peer review only - http://bmjopen.bmj.com/site/about/guidelines.xhtml

acute or chronic is insufficient. This is apparent when standard chronic pain definitions would group people with persistent mild symptoms with people who experience constant high levels of pain and other symptoms. Previous work has also highlighted problems defining acute and chronic pain,(25;29) but clinical guidelines are still formulated on this basis.(30;31) Researchers and clinicians should begin to rely less on standard definitions of back pain.

This study raises questions of when, during the life course, trajectory membership is determined. Adolescent trajectories of back pain showed some similar features to the current study (e.g. a cluster with very high probability of pain), whereas other trajectories indicated development of a pain condition.(32) Comparable trajectories were also identified for headache, facial pain and stomach pain in the adolescent cohort,(32) which indicates potential applicability of these findings to other conditions, particularly non-specific symptoms.(33;34)

Conclusions

We have provided unique evidence on the long-term course of back pain, and suggested a new framework for understanding the course of the condition. There is evidence against phases of change in back pain over time. There are some potential limitations of the study, but, if the results apply to a significant proportion of back pain patients, there are important clinical implications. First, a large proportion of those who do report initial pain recover quickly, but among those who do not, our results show that many will remain in the same trajectory over the longer-term. Second, if people in the most severe trajectories could be identified when seeking healthcare, they could be directed to specific targeted treatments. The

For peer review only - http://bmjopen.bmj.com/site/about/guidelines.xhtml

current study provides substantial new understanding of the long-term course of back pain, and has the potential to have impact in both research and clinical arenas.

For peer review only

For peer review only - http://bmjopen.bmj.com/site/about/guidelines.xhtml

Table 1. Monthly probability of experiencing each level of back pain based on cluster membership at 7-years

	Cluster (trajectory) from 7-year follow-up analysis			
	No / occasional pain	Persistent mild pain	Fluctuating pain	Persistent severe pain
Baseline				
No pain	0·87	0·15	0·01	0·00
Mild-moderate pain	0·13	0·80	0·51	0·21
High pain	0·00	0·05	0·48	0·79
Month 1				
No pain	0·85	0·06	0·00	0·00
Mild-moderate pain	0·15	0·91	0·62	0·17
High pain	0·00	0·04	0·38	0·83
Month 2				
No pain	0·65	0·06	0·00	0·00
Mild-moderate pain	0·35	0·89	0·12	0·11
High pain	0·00	0·05	0·88	0·89
Month 3				
No pain	0·70	0·07	0·01	0·00
Mild-moderate pain	0·30	0·86	0·58	0·17
High pain	0·00	0·07	0·42	0·83
Month 4				
No pain	0·66	0·09	0·00	0·00
Mild-moderate pain	0·34	0·88	0·29	0·18
High pain	0·00	0·03	0·71	0·82
Month 5				
No pain	0·75	0·26	0·19	0·00
Mild-moderate pain	0·25	0·69	0·73	0·02
High pain	0·00	0·05	0·09	0·98
Month 6				
No pain	0·80	0·16	0·02	0·00
Mild-moderate pain	0·20	0·79	0·66	0·11
High pain	0·00	0·05	0·32	0·89

For peer review only - http://bmjopen.bmj.com/site/about/guidelines.xhtml

Table 2. Cluster membership at 7 years stratified by original study cluster (n=155)

Original study cluster	No. in original study cluster	*n* (%)[a] in each cluster (trajectory) at 7 years			
		No or occasional pain	Persistent mild pain	Fluctuating pain	Persistent severe pain
Recovering	57	34 (59%)	18 (32%)	3 (5%)	2 (4%)
Persistent mild	51	12 (23%)	29 (56%)	8 (15%)	2 (5%)
Fluctuating	16	1 (7%)	6 (38%)	2 (13%)	7 (42%)
Severe chronic	31	0 (0%)	4 (12%)	4 (14%)	23 (74%)

[a] estimated following multiple imputation

Weighted kappa = 0.54 (95% CI 0.42, 0.65)

For peer review only - http://bmjopen.bmj.com/site/about/guidelines.xhtml

Table 3. Characteristics of cluster membership at 7-year baseline follow-up, Group 1 and 2 (n=155)

	Cluster (trajectory) from 7-year follow-up analysis				
	No or occasional pain	Persistent mild pain	Fluctuating pain	Persistent severe pain	p-value
% in cluster	31%	37%	11%	21%	
Age	46·3 (43·9, 48·6)	47·7 (45·5, 50·0)	46·3 (42·1, 50·6)	47·0 (43·7, 50·2)	0·85
Female	65% (51, 80)	63% (50, 77)	68% (43, 93)	63% (45, 81)	0·99
Pain intensity	0·8 (0, 1·8)	2·3 (1·8, 2·8)	4·9 (3·6, 6·3)	6·7 (5·8, 7·6)	<0·001
Leg pain	42% (26, 58)	51% (37, 65)	78% (54, 100)	83% (68, 98)	0·009
Upper body pain	52% (36, 68)	71% (58, 84)	88% (71, 100)	93% (84, 100)	0·004
Disability	2·0 (0, 4·1)	4·3 (3·0, 5·6)	8·7 (5·7, 11·7)	12·9 (10·5, 15·3)	<0·001
Anxiety	5·3 (4·1, 6·4)	6·8 (5·6, 8·0)	6·5 (4·4, 8·6)	8·8 (7·3, 10·3)	0·005
Depression	2·8 (1·8, 3·8)	4·9 (3·8, 6·0)	4·3 (2·8, 5·8)	7·4 (5·9, 8·8)	<0·001
PHQ 15	3·9 (2·6, 5·3)	5·0 (3·9, 6·1)	7·4 (4·3, 10·4)	7·7 (5·8, 9·7)	0·006
Insomnia	27% (12, 42)	42% (28, 57)	75% (51, 98)	80% (65, 96)	<0·001

Figures are mean (95% confidence interval) except female, leg pain, upper body pain and insomnia, which are percentage (95% confidence interval). PHQ-15 = Patient Health Questionnaire.

For peer review only

Table 4. Original study baseline characteristics of study participants

	Full 7-year follow-up (Group 1: n=112)	Limited 7-year follow-up (Group 2: n=43)	Groups 1 & 2: (n=155)	No 7-year follow-up data available (Group 3: n=187)	*p*-value: Groups 1&2 v. Group 3
Gender (female)†	72 (64%)	28 (65%)	100 (65%)	100 (53%)	0·04
Age (years)	46·9 (8·3)	47·0 (7·7)	47·0 (8·1)	47·4 (8·2)	0·63
Pain intensity	4·4 (2·7)	4·5 (2·9)	4·4 (2·8)	4·7 (2·5)	0·26
Disability	9·1 (6·8)	10·7 (6·8)	9·5 (6·8)	10·6 (6·4)	0·14
CPG IV†	30 (28%)	17 (40%)	47 (31%)	57 (32%)	0·86
Anxiety	8·2 (4.8)	9·1 (4·6)	8·5 (4·8)	8·6 (4·9)	0·82
Depression	6·1 (4·4)	8·4 (4·9)	6·8 (4·6)	7·5 (4·8)	0·15
Duration of pain†					
<= 6 months	42 (38%)	14 (33%)	56 (36%)	51 (28%)	0·10
7-35 months	23 (21%)	14 (33%)	37 (24%)	48 (26%)	
>= 3 years	46 (41%)	15 (35%)	61 (40%)	85 (46%)	
Cluster†					
Recovering	42 (38%)	15 (35%)	57 (37%)	47 (25%)	0·10
Persistent mild	34 (30%)	17 (40%)	51 (33%)	71 (38%)	
Fluctuating	13 (12%)	3 (7%)	16 (10%)	29 (16%)	
Severe-chronic	23 (21%)	8 (19%)	31 (20%)	40 (21%)	

Figures are mean (standard deviation) except those marked † which are numbers (percentage). CPG IV = Chronic Pain Grade IV

For peer review only

For peer review only - http://bmjopen.bmj.com/site/about/guidelines.xhtml

Figure 1. Trajectories of back pain intensity from original study and 7-year follow-up

For peer review only

For peer review only - http://bmjopen.bmj.com/site/about/guidelines.xhtml

Acknowledgements

We thank all the participants and general practices who participated in the original and follow-up phase of this study. We thank Professor Peter Croft for his comments on drafts of the paper.

Competing Interest statement

The authors have declared that no competing interests exist.

Funding statement

The work was supported by the Wellcome Trust grant number 083572. The funding body had no role in study design, data collection and analysis, decision to publish, or preparation of the manuscript.

Author's contributions

Kate Dunn conceived the study. All authors contributed to the design of the study. Paul Campbell and Kate Dunn coordinated the data collection. Kate Dunn and Kelvin Jordan analysed the data. All authors interpreted the data. Kate Dunn drafted the manuscript and all authors contributed to revisions. Kate Dunn had full access to all of the data in the study and takes responsibility for the integrity of the data and the accuracy of the data analysis. All authors approved the final version of the manuscript submitted for publication.

For peer review only

Checklist

STROBE statement enclosed.

Data sharing statement

The Arthritis Research UK Primary Care Centre has established data sharing arrangements to support joint publications and other research collaborations. Applications for access to anonymised data from our research databases are reviewed by the Centre's Data Custodian and Academic Proposal (DCAP) Committee and a decision regarding access to the data is made subject to the NRES ethical approval first provided for the study and to new analysis being proposed. Further information on our data sharing procedures can be found on the Centre’s website (http://www.keele.ac.uk/pchs/publications/datasharingresources/) or by emailing the Centre’s data manager (primarycare.datasharing@keele.ac.uk).

For peer review only

For peer review only - http://bmjopen.bmj.com/site/about/guidelines.xhtml

References

1. Vos T, Flaxman AD, Naghavi M, Lozano R, Michaud C et al. Years lived with disability (YLDs) for 1160 sequelae of 289 diseases and injuries 1990—2010: a systematic analysis for the Global Burden of Disease Study 2010. The Lancet 2012;**380**: 2163 – 2196.
2. Dunn KM, Croft PR. The importance of symptom duration in determining prognosis. Pain 2006;**121**:126-132.
3. Henschke N, Maher CG, Refshauge KM, Herbert RD, Cumming RG, Bleasel J et al. Prognosis in patients with recent onset low back pain in Australian primary care: inception cohort study. BMJ 2008;**337**:a171.
4. Dunn KM. Extending conceptual frameworks: life course epidemiology for the study of back pain. BMC Musculoskelet Disord 2010;**11**:23.
5. Costa LD, Maher CG, Hancock MJ, McAuley JH, Herbert RD, Costa LO. The prognosis of acute and persistent low-back pain: a meta-analysis. Canadian Medical Association Journal 2012;**84**:E613-E624.
6. Enthoven P, Skargren E, Carstensen J, Oberg B. Predictive factors for 1-year and 5-year outcome for disability in a working population of patients with low back pain treated in primary care. Pain 2006;**122**:137-144.
7. Burton AK, McClune TD, Clarke RD, Main CJ. Long-term follow-up of patients with low back pain attending for manipulative care-outcomes and predictors. Man Ther 2004;**9**:30-35.
8. Von Korff M, Miglioretti DL. A prognostic approach to defining chronic pain. Pain 2005;**117**:304-313.
9. Hill JC, Whitehurst DG, Lewis M, Bryan S, Dunn KM, Foster NE et al. Comparison of stratified primary care management for low back pain with current best practice (STarT Back): a randomised controlled trial. The Lancet 2011;**378**: 1560-1571.
10. Dunn KM, Jordan K, Croft PR. Characterising the course of low back pain: a latent class analysis. Am J Epidemiol 2006;**163**:754-761.
11. Raspe H, Huppe A, Matthis C. Theories and models of chronicity: on the way to a broader definition of chronic back pain. Schmerz 2003;**17**:359-366.
12. Laurens Rowe M. Backache at work. Fairport, New York: Perinton Press; 1983.
13. Dunn KM, Croft PR. Classification of low back pain in primary care: using "bothersomeness" to identify the most severe cases. Spine 2005;**30**:1887-1892.
14. Dunn KM, Jordan KP, Croft PR. Recall of medication use, self-care activities and pain intensity: a comparison of daily diaries and self-report questionnaires among low back pain patients. Primary Health Care Research & Development 2010;**11**:93-102.
15. Patrick DL, Deyo RA, Atlas SJ, Singer DE, Chapin AM, Keller RB. Assessing health-related quality of life in patients with sciatica. Spine 1995;**20**:1899-1908.

For peer review only - http://bmjopen.bmj.com/site/about/guidelines.xhtml

16. Dunn KM, Jordan K, Croft PR. Does questionnaire structure influence response in postal surveys? J Clin Epidemiol 2003;**56**:10-16.

17. Von Korff M, Ormel J, Keefe FJ, Dworkin SF. Grading the severity of chronic pain. Pain 1992;**50**:133-149.

18. Dunn KM, de Vet HCW, Hooper H, Ong BN, Croft PR. Measurement of back pain episode inception in questionnaires: a study combining quantitative and qualitative methods. Journal of Musculoskeletal Pain 2006;**14**:29-37.

19. Zigmond AS, Snaith RP. The Hospital Anxiety and Depression Scale. Acta Psychiatr Scand 1983;**67**:361-370.

20. Jenkins CD, Stanton BA, Niemcryk SJ, Rose RM. A scale for the estimation of sleep problems in clinical research. J Clin Epidemiol 1988;**41**:313-321.

21. Morphy H, Dunn KM, Lewis M, Boardman HF, Croft PR. Epidemiology of insomnia: a longitudinal study in a UK population. Sleep 2007;**30**:274-280.

22. Kroenke K, Spitzer RL, Williams JB. The PHQ-15: validity of a new measure for evaluating the severity of somatic symptoms. Psychosom Med 2002;**64**:258-266.

23. Waddell G. The Back Pain Revolution. Edinburgh: Churchill Livingstone; 1998.

24. Hestbaek L, Leboeuf-Yde C, Manniche C. Low back pain: what is the long-term course? A review of studies of general patient populations. Eur Spine J 2003;**12**:149-165.

25. Young AE, Wasiak R, Phillips L, Gross DP. Workers' perspectives on low back pain recurrence: "It comes and goes and comes and goes, but it's always there". Pain 2011;**152**:204-211.

26. Tamcan O, Mannion AF, Eisenring C, Horisberger B, Elfering A, Müller U. The course of chronic and recurrent low back pain in the general population. Pain 2010;**150**:451-457.

27. Raspe H. (2010) Measuring the impact of chronic pain on populations: a narrative review. In: Croft P, Blyth FM, van der Windt D, editors. Chronic pain epidemiology: From aetiology to public health. 1st ed. Oxford: Oxford University Press. pp. 61-70.

28. Hill JC, Vohora K, Dunn KM, Main CJ, Hay E. Comparing the STarT Back screening tool's subgroup allocation of individual patients with that of independent clinical experts. Clin J Pain 2010;**26**: 783-787.

29. Von Korff M, Dunn KM. Chronic pain reconsidered. Pain 2008;**138**:267-276.

30. Savigny P, Kuntze S, Watson P, Underwood M, Ritchie G, Cotterell M. (2009) Early management of persistent non-specific low back pain. London, National Collaborating Centre for Primary Care and Royal College of General Practitioners.

31. Chou R, Qaseem A, Snow V, Casey D, Cross JT, Jr., Shekelle P et al. Diagnosis and treatment of low back pain: a joint clinical practice guideline from the American College of Physicians and the American Pain Society. Ann Intern Med 2007;**147**:478-491.

32. Dunn KM, Jordan KP, Mancl L, Drangsholt MT, Le Resche L. Trajectories of pain in adolescents: a prospective cohort study. Pain 2011;**152**:66-73.

For peer review only - http://bmjopen.bmj.com/site/about/guidelines.xhtml

For peer review only

33. Aggarwal VR, McBeth J, Zakrzewska JM, Lunt M, Macfarlane GJ. The epidemiology of chronic syndromes that are frequently unexplained: do they have common associated factors? Int J Epidemiol 2006;**35**:468-476.

34. Nimnuan C, Rabe-Hesketh S, Wessely S, Hotopf M. How many functional somatic syndromes? J Psychosom Res 2001;**51**:549-557.

For peer review only

For peer review only - http://bmjopen.bmj.com/site/about/guidelines.xhtml

Appendix

Latent class analysis

The assumption behind the longitudinal latent class analysis was that there exists distinct pathways of back pain, and hence the participants can be grouped into distinct clusters (known as latent classes) based on their profile of back pain over the 6-months, with each subject belonging to one cluster. Specifically, longitudinal latent class analysis aims to obtain the smallest number of clusters that accounts for the associations between the monthly pain levels.

Latent class models were fitted successively, starting with a one cluster model and then sequentially adding another cluster for each successive model. There is no gold standard goodness of fit criteria for longitudinal latent class analysis models and so the final number of clusters was determined by examining the optimal models based on each of Akaike's Information criterion (AIC, and revised version AIC3), Bayes information criterion and the Consistent Akaike's Information criterion (1). The optimal number of clusters for each criterion is where the information criterion value is at its lowest. The percentage reduction in the model fit likelihood ratio chi-squared statistic (L^2) from the model with one cluster, was also calculated, with the optimal number of clusters where the percentage reduction is considered minor. The resultant optimal models were then compared on size (clusters should include at least 10% of participants) and with regards to having distinct cluster characteristics to determine the final number of clusters. LatentGold version 4.0 was used to perform the analyses. LatentGold uses both the EM and Newton-Raphson algorithms to estimate model parameters. 1000 different random starting values were used, each of which included 100 iterations. The bivariate residuals were used to assess violation of the local independence assumption for the optimal model. Local independence means that within clusters the

For peer review only - http://bmjopen.bmj.com/site/about/guidelines.xhtml

probability of a certain level of pain for any month is not related to the level of pain for any other month. Restricted latent class analysis models to address any violation were developed where the bivariate residuals between monthly pain ratings was greater than the recommended level of 1 (2).

The goodness of fit statistics for the longitudinal latent class analysis are shown in the Appendix Table 1. Between 3 and 6 clusters were considered optimal by the different goodness of fit measures, but the 6 cluster model included a cluster with only 2% of participants and so was dropped from consideration. A restricted 4 cluster model was ultimately selected as optimal, as the clusters were distinct, and included at least 10% of participants in each cluster. The fifth cluster in the 5-cluster model was a subgroup of a cluster in the 4-cluster model and did not have distinct characteristics.

An alternative to longitudinal latent class analysis which explicitly takes the time order into account is latent class growth analysis. Derivation of clusters using latent class growth analysis (not shown here) yielded similar trajectories presumably due to the relative stability of pain in participants. The pain profiles of individuals in each longitudinal latent class analysis cluster matched that of the cluster as a whole, and the clusters themselves revealed distinct pathways of pain and related health status. Some of the health status measures exhibited some skewness in scores but analysis comparing median and interquartile range scores showed the same patterns across clusters and led to the same conclusions as for the main analysis.

For peer review only - http://bmjopen.bmj.com/site/about/guidelines.xhtml

Appendix Table 1. Goodness of fit statistics for the longitudinal latent class analysis

Model	L^2	% reduction in L^2 from H_0	AIC_{LL}	$AIC3_{LL}$	BIC_{LL}	$CAIC_{LL}$
1 Cluster (H_0)	1150·56	-	1759·65	1773·65	1800·43	1814·43
2 Cluster	748·35	35	1373·44	1395·44	1437·52	1459·52
3 Cluster	556·74	52	1197·83	1227·83	1285·21[a]	1315·21[a]
4 Cluster	528·27	54	1185·36	1223·36	1296·04	1334·04
5 Cluster	501·80	56	1174·90	1220·90	1308·89	1354·88
6 Cluster	476·81	59	1165·90[a]	1219·90[a]	1323·19	1377·19
7 Cluster	461·89	60	1166·98	1228·98	1347·56	1409·56
8 Cluster	447·62	61	1168·71	1238·71	1372·60	1442·60

[a] optimal unrestricted model for that goodness of fit statistic

AIC= Akaike's Information criterion; BIC= Bayes information criterion; CAIC= Consistent Akaike's Information criterion.

For peer review only - http://bmjopen.bmj.com/site/about/guidelines.xhtml

Appendix References

(1) Ware JE, Kolinski M, Keller.S.D. How to score the SF-12 Physical and Mental Health Summaries: A User's Manual. Boston, MA: The Health Institute, New England Medical Centre; 1995.

(2) Magidson J, Vermunt JK. Latent Class Models. In: Kaplan DW, ed. The Sage Handbook of Quantitative Methodology for the Social Sciences. Thousand Oaks, CA: Sage Publications; 2004: 175-98

For peer review only

Title: Long-term trajectories of back pain: cohort study with seven year follow-up

Authors: Kate M Dunn*, Paul Campbell*, Kelvin P Jordan*

Author affiliations

*Arthritis Research UK Primary Care Centre, Primary Care Sciences, Keele University, Staffordshire, ST5 5BG, UK.

Corresponding Authors Details

Dr Kate M Dunn, Arthritis Research UK Primary Care Centre, Primary Care Sciences, Keele University, Staffordshire, ST5 5BG, UK.

Tel: + 44 (0) 1782 734703; Fax: + 44 (0) 1782 733911, Email: k.m.dunn@keele.ac.uk

http://www.keele.ac.uk/research/pchs/pcmrc/

For peer review only

Keywords: Low Back Pain; Prospective Studies; Cluster Analysis; Longitudinal Studies; Pain Measurement.

Word count: ~~2560~~2767.

For peer review only - http://bmjopen.bmj.com/site/about/guidelines.xhtml

Abstract

Objective: To describe long-term trajectories of back pain.

Design: Monthly data collection for 6-months at 7-year follow-up of participants in a prospective cohort study.

Setting: Primary care practices in Staffordshire, UK.

Participants: 228 people consulting their GP with back pain, on whom information on 6-month back pain trajectories had been collected during 2001-3, and who had valid consent and contact details in 2009-10, were contacted. 155 participants (68% of those contacted) responded and provided sufficient data for primary analyses.

Outcome measures: Trajectories based on patients' self-reports of back pain, identified using Longitudinal Latent Class Analysis. Trajectories characterised using information on disability, psychological status and presence of other symptoms.

Results: Four clusters with different back pain trajectories at follow-up were identified: (i) no or occasional mild pain, (ii) persistent mild pain, (iii) fluctuating pain, and (iv) persistent severe pain. Trajectory clusters differed significantly from each other in terms of disability, psychological status and other symptoms. Most participants remained in a similar trajectory as seven years previously (weighted kappa 0.54; 95% CI 0.42, 0.65).

Conclusions: Most people with back pain appear to follow a particular pain trajectory over long time periods, and do not have frequently recurring or widely fluctuating patterns. Results are limited by lack of information about the time between data collection periods, and by loss to follow-up. However, fFindings do raise questions about standard divisions into acute and chronic back pain. A new framework for understanding the course of back pain is proposed.

For peer review only

For peer review only - http://bmjopen.bmj.com/site/about/guidelines.xhtml

Article Summary

Article focus

- Most research studies have limited follow-up in terms of frequency of data collection and long-term timing
- Previous work has used frequent data collection points to identify new short-term trajectories of back pain
- This study aimed to carry out long-term follow-up of people in those trajectories to identify the long-term course and trajectories of back pain.

Key messages

- Four clusters with different back pain trajectories and characteristics at follow-up were identified: (i) no or occasional ~~mild~~ pain, (ii) persistent mild pain, (iii) fluctuating pain, and (iv) persistent severe pain.
- Most participants remained in a similar trajectory as seven years previously, indicating that people with back pain follow a particular pain trajectory over long time periods.
- Findings raise questions about standard divisions into acute and chronic back pain based purely on duration of current episode.

Strengths and limitations of this study

- The study benefits from long-term follow-up, prospective design, frequent follow-up during study periods, robust analyses and use of validated questionnaire instruments.
- The study was limited by loss to follow-up, meaning restricted numbers for full analysis, but multiple imputation was used to investigate the implications of this.
- Data collection phases were 7-years apart, and similar information about trajectories in the interim period is unavailable.

For peer review only

For peer review only - http://bmjopen.bmj.com/site/about/guidelines.xhtml

Introduction

Back pain is common – it has been recently highlighted as the single leading cause of years lived with disability worldwide (1) and many people experience pain over long periods. Among primary care consulters, 38% report having their symptoms for over three years.(2) Even among people in primary care with acute back pain, 75% report previous back pain,(3) indicating that even if not constantly present, back pain is a long-term experience. This has led to a suggestion to use a longer-term, lifecourse approach to studying back pain.(4)

The long-term experience of back pain is often not addressed by researchers. In a recent review of back pain prognosis, only 1 of the 33 included studies had follow-up beyond a year.(5) Studies with shorter term follow-up can only represent a compressed view of the long-term pain experience. The few longer-term studies have limited numbers of follow-up points,(6,7,8) Knowledge of prognosis is important, as stratifying back pain management based on risk of poor prognosis can be clinically and cost-effective,(9) with benefits for targeting early treatment and referrals. However previous research is unable to fully reflect the detailed course of back pain over time, or inform about long-term prognosis.

In 2001-2 we studied a cohort of people consulting in primary care with back pain.(10) We identified four distinct clusters of people with different trajectories: (i) recovering, (ii) persistent mild, (iii) fluctuating pain, (iv) severe chronic back pain. Duration of back pain at baseline increased with rising severity of trajectory, potentially indicating phases of increasing severity in the long-term course. This is supported by models of stages of back

For peer review only - http://bmjopen.bmj.com/site/about/guidelines.xhtml

pain chronicity (11) and degeneration with age.(12) Alternatively, trajectories could represent distinct groups with stable long-term pain. We aimed to describe long-term trajectories of back pain through ~~the seven year~~ a 6 month follow-up period of a cohort of back pain patients previously studied seven years earlier.

Methods

This is a follow-up of participants in a back pain cohort study whose short term (6 month) back pain trajectories had been derived in 2001-2.(10)

Study participants

The original study identified people aged 30-59 years consulting with back pain at one of five general practices in North Staffordshire, UK, during 2001-2. Details are published elsewhere.(13) Briefly, participants returning baseline questionnaires and consenting to follow-up were sent monthly questionnaires. Those returning four or more questionnaires during the first six months were included in a longitudinal latent class analysis to determine trajectories of back pain.(10) Of the 342 participants in this original analysis, 73% (n=250) gave their consent to be contacted again. In 2009, current contact details were not available for 22 (6%), leaving 228 people from the original analysis invited to take part at seven year follow-up.

For peer review only

For peer review only - http://bmjopen.bmj.com/site/about/guidelines.xhtml

Data collection at seven years

Self-completion questionnaires were mailed to the 228 study participants (seven year baseline mailing) with reminders at two and four weeks, and brief questionnaires for non-responders at six weeks. Participants giving informed consent were sent brief monthly questionnaires for six months (the same data collection technique as the original study).

All questionnaires contained the same key measures. Pain intensity was measured using the mean of three 0-10 numerical rating scales.(14) Disability was measured using the modified 23-item Roland-Morris Disability Questionnaire (RMDQ).(15) These instruments were used in the original study,(10) and there is evidence of reliability in UK primary care back pain patients.(16) The Chronic Pain Grade classified individuals into grades of chronic pain;(17) this was included in the brief seven year baseline mailing for non-responders. Back pain duration was recalled time since the last pain-free month.(18)

The Hospital Anxiety and Depression Scale (HADS) was used to assess psychological status.(19) It produces scores from 0-21, with higher scores indicating more severe symptoms. Insomnia was defined as reporting having trouble falling or staying asleep, waking up several times a night, or waking up feeling tired on most nights.(20) This definition has been used previously in pain samples.(21) Somatic symptoms were measured using the 15-item Patient Health Questionnaire (PHQ-15) (22) which is scored from 0 (not bothered with any symptoms) to 30 (bothered a lot with 15 symptoms). Leg pain was self-

For peer review only - http://bmjopen.bmj.com/site/about/guidelines.xhtml

For peer review only

reported pain travelling from the back to the leg(s), and upper body pain was self-reported pain in the shoulder, arm, neck or head, during the previous two weeks.

Analysis

Two primary analysis groups were formed from responders to this seven-year follow-up study. Group one participants returned the seven year baseline questionnaire plus three or more questionnaires from months one to six. Group two included participants with insufficient seven year follow-up data for full analyses, but who provided adequate information for multiple imputation to be carried out.

For Group one participants, monthly back pain intensity scores were trichotomized into no pain (scoring less than one), mild-moderate pain, and high pain (scored five or more). Longitudinal latent class analysis was used to group participants into clusters based on the trajectory of their back pain over these six months as in the original study.(10) In longitudinal latent class analysis, each participant is allocated to the cluster best matching their pain profile, based on each participant's probability of belonging to each cluster, with participants allocated to the cluster for which they have the largest probability. Participants should be clearly assigned to a single cluster with high probability. Cluster-specific probabilities of having each level of pain for each month, given membership of that cluster, allow development of pain pathways for each cluster. See appendix for more details.

For peer review only - http://bmjopen.bmj.com/site/about/guidelines.xhtml

For Group two participants, the multiple imputation procedure in Stata/IC v11.1 software with 50 imputations, through a multinomial logistic regression, was used to impute membership of the seven year clusters identified for Group one. Information on cluster from the original study, plus outcome measures from the seven year baseline questionnaire were used to impute cluster membership.

Membership of clusters from both study phases (original and seven year follow-up) were compared to investigate long-term patterns of trajectory membership. Stability of cluster membership was assessed using weighted kappa. Kappa can be interpreted as agreement (stability) between original and seven year follow-up cluster memberships beyond chance, with values of 1 indicating perfect agreement and 0 indicating agreement no better than chance. The seven year derived clusters (actual or imputed) were compared on the key measures of the seven year baseline questionnaire, using simple linear or logistic regression as appropriate through the multiple imputation estimate commands in Stata/IC v11.1.

In order to address potential issues from loss to follow-up from the original 2001-3 trajectories analysis, an additional Group three was formed. This included everyone from the original analysis who was not included in the primary analysis at 7 years (above): seven-year responders who provided insufficient data, non-responders at seven years, people who could not be traced, and those not giving consent to follow-up. Groups one and two combined were compared to Group three on baseline demographic, pain, anxiety and depression from the original study using t-tests or chi-squared tests as appropriate.

For peer review only - http://bmjopen.bmj.com/site/about/guidelines.xhtml

As sensitivity analysis, seven year cluster membership was imputed for Group two and Group three participants using information from the original study (baseline Roland-Morris Disability Questionnaire, Chronic Pain Grade, pain duration and original longitudinal latent class analysis cluster). Comparisons between the original cluster and seven year actual or imputed cluster membership for participants across all three groups were performed.

Ethics Statement

The original study and the 2009-10 follow-up phase were independently approved by North Staffordshire Local Research Ethics Committee and South Staffordshire Research Ethics Committee respectively.

Results

Primary analyses were carried out on 155 responders (68% of the 228 contacted): 112 in Group one (full data available) and 43 in Group two (imputation required).

Clusters at seven year follow-up

The optimal number of clusters resulting from longitudinal latent class analysis was four (see appendix). 84% of Group one participants had an average probability of greater than 0.90 of

For peer review only

being allocated to their assigned cluster, indicating distinct classification. Group two participants were allocated to these clusters using multiple imputation.

The estimated probability of monthly levels of pain within clusters is shown in Table 1. These monthly probabilities of pain can be interpreted to describe the occurrence of pain, for example, a probability of mild-moderate pain of 0.13 at baseline for the first Cluster indicates that one in every eight people in that group are likely to have experienced mild-moderate pain that month. The first cluster identified (31% of Group one and Group two) mostly had no pain (estimated monthly probabilities of no pain 0.65-0.87), with occasional mild episodes (cluster labelled 'no or occasional pain'). Participants in this cluster generally reported no pain on at least 4 occasions over the six months and did not report high pain. The second cluster (37%) had mild pain intensity throughoutmost of the time, with a maximum of 1-2 months of no pain; only 17% of the cluster ever reported high pain. Their, with monthly probabilities of mild pain were between 0.69-0.91 ('persistent mild pain'). The third cluster (11%) had pain fluctuating between mild and high levels ('fluctuating pain'), and rarely reported no pain. The final cluster (21%) had high pain intensity levels throughout, with monthly probabilities of high pain between 0.79-0.98 ('persistent severe pain'), and never reported no pain.

Comparison of clusters from original study and seven year follow-up

The identified trajectories of back pain intensity for the original study and the seven year follow-up are illustrated in Figure 1.

For peer review only - http://bmjopen.bmj.com/site/about/guidelines.xhtml

Most participants stayed in a similar cluster between the two study phases (weighted kappa 0.54 (95% confidence interval (CI) 0.42, 0.65)) (Table 2). 74% (95% CI 57%, 92%) of those originally in the most severe trajectory remained in an equivalent cluster at seven years. Over half the participants in the two mildest clusters in the original study (recovering: 59%; 95% CI 44%, 74%; persistent mild pain: 56%; 95% CI 40%, 73%) stayed in the most comparable trajectory at seven years, and most who changed moved to the other mild trajectory. The fluctuating group in the original study (the smallest group) did not show a stable pattern, with 87% of participants changing cluster, mainly to persistent mild or persistent severe clusters.

Pain intensity, disability and psychological status all differed significantly between the seven year trajectories, with the no or occasional pain cluster having the lowest disability levels (mean RMDQ score 2.0), least pain intensity (mean 0.8) and best psychological status (mean HADS depression score 2.8), and the persistent severe pain cluster having the highest disability (mean RMDQ score 12.9), worst pain intensity (mean 6.7) and poorest psychological status (mean HADS depression score 7.4) (Table 3). Similar statistically significant differences were also present in the original study.(10) The clusters also differed significantly in terms of the presence of somatic symptoms and insomnia, with the mean symptom score (PHQ-15) ranging from 3.9 in the no or occasional pain group to 7.7 in the persistent severe pain cluster, and the proportion classified with insomnia ranging from 27% to 80%.

Sensitivity analyses

For peer review only - http://bmjopen.bmj.com/site/about/guidelines.xhtml

Group three comprised 25 seven-year responders who provided insufficient data, 48 non-responders at seven years, plus the people from the original study who did not give consent to follow-up (n=92) or could not be traced (n=22). Original study baseline characteristics of the three Groups are shown in Table 4. The only significant difference between participants in Groups one and two and those in Group three was gender, with fewer females in Group three (p=0.04).

Including imputed data from Group three participants as well as Group two made little difference to the estimated relative sizes of the seven year clusters reported above, and gave similar patterns of disability, psychological status and other symptoms.

For peer review only

Discussion

This study provides unique prospective data on the long-term course of back pain. It suggests that most people remain in a particular pain trajectory, with similar characteristics, when estimated across a seven year period. These findings do not support the hypotheses that there are phases, or degeneration, in the course of back pain over time. Our findings show that widely fluctuating pain is not common (the fluctuating cluster was consistently smallest), and most people have pain patterns varying slightly around their own mean long-term pain. This includes people who recover quickly, and maintain very low (or no) pain, and people who have persistently higher levels of pain. Descriptions of back pain often assume a prevailing pattern of recurrent or fluctuating pain.(23;24) Our findings, and recent qualitative work,(25) provide evidence that these opinions ~~are~~ do not give the full picture. However, our study reports pain trajectories among individuals who have sought healthcare, and although recent work identifying general population trajectories of back pain showed trajectories similar to ours,(26) their fluctuating cluster comprised more of the population (35%).

Strengths of the current study include the long-term nature, prospective design, frequent follow-up during study periods, robust analyses and use of validated questionnaire instruments. However, the study did suffer from loss to follow-up, meaning limited numbers for full analysis. Multiple imputation was used to investigate the implications of this, and participants included in primary analyses were similar to those excluded, but the possibility of selection bias and residual confounding cannot be ruled out. Although this study had frequent follow-up points, data collection phases were 7-years apart, and similar information about trajectories in the interim period is unavailable.

For peer review only - http://bmjopen.bmj.com/site/about/guidelines.xhtml

Few studies have suggested models for long-term change in back pain. Our study gives some support to the model by Raspe et al,(11) as worsening back pain trajectory was significantly associated with more disability, distress, other pains and symptoms, similar to their model of symptom 'amplification'. However, the prospective nature of our study indicates that this 'amplification' is not related to deterioration over time or stages of change, but describes underlying differences between groups of people whose general pattern of pain does not appear to change over time. In addition, it appears that the spread of pain, further complaints and depressive symptoms increases fairly consistently with increasing severity of pain trajectory, rather than occurring in discrete stages, as in the amplification model.(11;27) Our results also do not support models of degeneration with age,(12) as clusters do not differ by age. Our findings suggest a new framework model for the long-term course of back pain, comprising four different types of back pain trajectory, each with characteristic pain patterns, disability levels, psychological status and wider symptoms.

New research is emerging on the treatment of back pain according to prognostic risk groups,(9) but questions have been raised about timing of risk group allocation.(28) Our research highlights potentially stable groups of people with different pain trajectories and characteristics. Comparison of the two study phases showed that no cluster changed mean Roland-Morris Disability Questionnaire score by over 2.5 points (a recommended clinically important change for back pain). This knowledge could improve allocation of treatment according to prognostic risk. However, collecting data over six months to allocate treatment is not clinically plausible, and work is needed to identify pain trajectories concisely and accurately. An important implication of our findings is that classifying back pain simply as

For peer review only - http://bmjopen.bmj.com/site/about/guidelines.xhtml

acute or chronic is insufficient. This is apparent when standard chronic pain definitions would group people with persistent mild symptoms with people who experience constant high levels of pain and other symptoms. Previous work has also highlighted problems defining acute and chronic pain,(25;29) but clinical guidelines are still formulated on this basis.(30;31) Researchers and clinicians should begin to rely less on standard definitions of back pain.

This study raises questions of when, during the life course, trajectory membership is determined. Adolescent trajectories of back pain showed some similar features to the current study (e.g. a cluster with very high probability of pain), whereas other trajectories indicated development of a pain condition.(32) Comparable trajectories were also identified for headache, facial pain and stomach pain in the adolescent cohort,(32) which indicates potential applicability of these findings to other conditions, particularly non-specific symptoms.(33;34)

Conclusions

We have provided unique evidence on the long-term course of back pain, and suggested a new framework for understanding the course of the condition. There is evidence against phases of change in back pain over time. There are some potential limitations of the study, but, if the results apply to a significant proportion of back pain patients, there are important clinical implications. First, a large proportion of those who do report initial pain recover quickly, but among those who do not, our results show that many will remain in the same trajectory over the longer-term. Second, if people in the most severe trajectories could be identified when seeking healthcare, they could be directed to specific targeted treatments. The

For peer review only - http://bmjopen.bmj.com/site/about/guidelines.xhtml

current study provides substantial new understanding of the long-term course of back pain, and has the potential to have impact in both research and clinical arenas.

For peer review only

For peer review only - http://bmjopen.bmj.com/site/about/guidelines.xhtml

Table 1. Monthly probability of experiencing each level of back pain based on cluster membership at 7-years

	Cluster (trajectory) from 7-year follow-up analysis			
	No / occasional pain	Persistent mild pain	Fluctuating pain	Persistent severe pain
**Baseline**				
No pain	0·87	0·15	0·01	0·00
Mild-moderate pain	0·13	0·80	0·51	0·21
High pain	0·00	0·05	0·48	0·79
**Month 1**				
No pain	0·85	0·06	0·00	0·00
Mild-moderate pain	0·15	0·91	0·62	0·17
High pain	0·00	0·04	0·38	0·83
**Month 2**				
No pain	0·65	0·06	0·00	0·00
Mild-moderate pain	0·35	0·89	0·12	0·11
High pain	0·00	0·05	0·88	0·89
**Month 3**				
No pain	0·70	0·07	0·01	0·00
Mild-moderate pain	0·30	0·86	0·58	0·17
High pain	0·00	0·07	0·42	0·83
**Month 4**				
No pain	0·66	0·09	0·00	0·00
Mild-moderate pain	0·34	0·88	0·29	0·18
High pain	0·00	0·03	0·71	0·82
**Month 5**				
No pain	0·75	0·26	0·19	0·00
Mild-moderate pain	0·25	0·69	0·73	0·02
High pain	0·00	0·05	0·09	0·98
**Month 6**				
No pain	0·80	0·16	0·02	0·00
Mild-moderate pain	0·20	0·79	0·66	0·11
High pain	0·00	0·05	0·32	0·89

For peer review only

For peer review only - http://bmjopen.bmj.com/site/about/guidelines.xhtml

Table 2. Cluster membership at 7 years stratified by original study cluster (n=155)

Original study cluster	No. in original study cluster	*n* (%)[a] in each cluster (trajectory) at 7 years			
		No or occasional pain	Persistent mild pain	Fluctuating pain	Persistent severe pain
Recovering	57	34 (59%)	18 (32%)	3 (5%)	2 (4%)
Persistent mild	51	12 (23%)	29 (56%)	8 (15%)	2 (5%)
Fluctuating	16	1 (7%)	6 (38%)	2 (13%)	7 (42%)
Severe chronic	31	0 (0%)	4 (12%)	4 (14%)	23 (74%)

[a] estimated following multiple imputation

Weighted kappa = 0.54 (95% CI 0.42, 0.65)

For peer review only - http://bmjopen.bmj.com/site/about/guidelines.xhtml

For peer review only

Table 3. Characteristics of cluster membership at 7-year baseline follow-up, Group 1 and 2 (n=155)

	Cluster (trajectory) from 7-year follow-up analysis				
	No or occasional pain	Persistent mild pain	Fluctuating pain	Persistent severe pain	p-value
% in cluster	31%	37%	11%	21%	
Age	46·3 (43·9, 48·6)	47·7 (45·5, 50·0)	46·3 (42·1, 50·6)	47·0 (43·7, 50·2)	0·85
Female	65% (51, 80)	63% (50, 77)	68% (43, 93)	63% (45, 81)	0·99
Pain intensity	0·8 (0, 1·8)	2·3 (1·8, 2·8)	4·9 (3·6, 6·3)	6·7 (5·8, 7·6)	<0·001
Leg pain	42% (26, 58)	51% (37, 65)	78% (54, 100)	83% (68, 98)	0·009
Upper body pain	52% (36, 68)	71% (58, 84)	88% (71, 100)	93% (84, 100)	0·004
Disability	2·0 (0, 4·1)	4·3 (3·0, 5·6)	8·7 (5·7, 11·7)	12·9 (10·5, 15·3)	<0·001
Anxiety	5·3 (4·1, 6·4)	6·8 (5·6, 8·0)	6·5 (4·4, 8·6)	8·8 (7·3, 10·3)	0·005
Depression	2·8 (1·8, 3·8)	4·9 (3·8, 6·0)	4·3 (2·8, 5·8)	7·4 (5·9, 8·8)	<0·001
PHQ 15	3·9 (2·6, 5·3)	5·0 (3·9, 6·1)	7·4 (4·3, 10·4)	7·7 (5·8, 9·7)	0·006
Insomnia	27% (12, 42)	42% (28, 57)	75% (51, 98)	80% (65, 96)	<0·001

Figures are mean (95% confidence interval) except female, leg pain, upper body pain and insomnia, which are percentage (95% confidence interval). PHQ-15 = Patient Health Questionnaire.

For peer review only

For peer review only - http://bmjopen.bmj.com/site/about/guidelines.xhtml

Table 4. Original study baseline characteristics of study participants

	Full 7-year follow-up (Group 1: n=112)	Limited 7-year follow-up (Group 2: n=43)	Groups 1 & 2: (n=155)	No 7-year follow-up data available (Group 3: n=187)	*p*-value: Groups 1&2 v. Group 3
Gender (female)†	72 (64%)	28 (65%)	100 (65%)	100 (53%)	0·04
Age (years)	46·9 (8·3)	47·0 (7·7)	47·0 (8·1)	47·4 (8·2)	0·63
Pain intensity	4·4 (2·7)	4·5 (2·9)	4·4 (2·8)	4·7 (2·5)	0·26
Disability	9·1 (6·8)	10·7 (6·8)	9·5 (6·8)	10·6 (6·4)	0·14
CPG IV†	30 (28%)	17 (40%)	47 (31%)	57 (32%)	0·86
Anxiety	8·2 (4.8)	9·1 (4·6)	8·5 (4·8)	8·6 (4·9)	0·82
Depression	6·1 (4·4)	8·4 (4·9)	6·8 (4·6)	7·5 (4·8)	0·15
Duration of pain†					
<= 6 months	42 (38%)	14 (33%)	56 (36%)	51 (28%)	0·10
7-35 months	23 (21%)	14 (33%)	37 (24%)	48 (26%)	
>= 3 years	46 (41%)	15 (35%)	61 (40%)	85 (46%)	
Cluster†					
Recovering	42 (38%)	15 (35%)	57 (37%)	47 (25%)	0·10
Persistent mild	34 (30%)	17 (40%)	51 (33%)	71 (38%)	
Fluctuating	13 (12%)	3 (7%)	16 (10%)	29 (16%)	
Severe-chronic	23 (21%)	8 (19%)	31 (20%)	40 (21%)	

Figures are mean (standard deviation) except those marked † which are numbers (percentage). CPG IV = Chronic Pain Grade IV

For peer review only - http://bmjopen.bmj.com/site/about/guidelines.xhtml

Figure 1. Trajectories of back pain intensity from original study and 7-year follow-up

For peer review only

For peer review only - http://bmjopen.bmj.com/site/about/guidelines.xhtml

Acknowledgements

We thank all the participants and general practices who participated in the original and follow-up phase of this study. We thank Professor Peter Croft for his comments on drafts of the paper.

Competing Interest statement

The authors have declared that no competing interests exist.

Funding statement

The work was supported by the Wellcome Trust grant number 083572. The funding body had no role in study design, data collection and analysis, decision to publish, or preparation of the manuscript.

Author's contributions

Kate Dunn conceived the study. All authors contributed to the design of the study. Paul Campbell and Kate Dunn coordinated the data collection. Kate Dunn and Kelvin Jordan analysed the data. All authors interpreted the data. Kate Dunn drafted the manuscript and all authors contributed to revisions. Kate Dunn had full access to all of the data in the study and takes responsibility for the integrity of the data and the accuracy of the data analysis. All authors approved the final version of the manuscript submitted for publication.

For peer review only - http://bmjopen.bmj.com/site/about/guidelines.xhtml

For peer review only

Checklist

STROBE statement enclosed.

Data sharing statement

The Arthritis Research UK Primary Care Centre has established data sharing arrangements to support joint publications and other research collaborations. Applications for access to anonymised data from our research databases are reviewed by the Centre's Data Custodian and Academic Proposal (DCAP) Committee and a decision regarding access to the data is made subject to the NRES ethical approval first provided for the study and to new analysis being proposed. Further information on our data sharing procedures can be found on the Centre’s website (http://www.keele.ac.uk/pchs/publications/datasharingresources/) or by emailing the Centre’s data manager (primarycare.datasharing@keele.ac.uk).

For peer review only

References

1. Vos T, Flaxman AD, Naghavi M, Lozano R, Michaud C et al. Years lived with disability (YLDs) for 1160 sequelae of 289 diseases and injuries 1990—2010: a systematic analysis for the Global Burden of Disease Study 2010. The Lancet 2012;**380**: 2163 – 2196.
2. Dunn KM, Croft PR. The importance of symptom duration in determining prognosis. Pain 2006;**121**:126-132.
3. Henschke N, Maher CG, Refshauge KM, Herbert RD, Cumming RG, Bleasel J et al. Prognosis in patients with recent onset low back pain in Australian primary care: inception cohort study. BMJ 2008;**337**:a171.
4. Dunn KM. Extending conceptual frameworks: life course epidemiology for the study of back pain. BMC Musculoskelet Disord 2010;**11**:23.
5. Costa LD, Maher CG, Hancock MJ, McAuley JH, Herbert RD, Costa LO. The prognosis of acute and persistent low-back pain: a meta-analysis. Canadian Medical Association Journal 2012;**84**:E613-E624.
6. Enthoven P, Skargren E, Carstensen J, Oberg B. Predictive factors for 1-year and 5-year outcome for disability in a working population of patients with low back pain treated in primary care. Pain 2006;**122**:137-144.
7. Burton AK, McClune TD, Clarke RD, Main CJ. Long-term follow-up of patients with low back pain attending for manipulative care-outcomes and predictors. Man Ther 2004;**9**:30-35.
8. Von Korff M, Miglioretti DL. A prognostic approach to defining chronic pain. Pain 2005;**117**:304-313.
9. Hill JC, Whitehurst DG, Lewis M, Bryan S, Dunn KM, Foster NE et al. Comparison of stratified primary care management for low back pain with current best practice (STarT Back): a randomised controlled trial. The Lancet 2011;**378**: 1560-1571.
10. Dunn KM, Jordan K, Croft PR. Characterising the course of low back pain: a latent class analysis. Am J Epidemiol 2006;**163**:754-761.
11. Raspe H, Huppe A, Matthis C. Theories and models of chronicity: on the way to a broader definition of chronic back pain. Schmerz 2003;**17**:359-366.
12. Laurens Rowe M. Backache at work. Fairport, New York: Perinton Press; 1983.
13. Dunn KM, Croft PR. Classification of low back pain in primary care: using "bothersomeness" to identify the most severe cases. Spine 2005;**30**:1887-1892.
14. Dunn KM, Jordan KP, Croft PR. Recall of medication use, self-care activities and pain intensity: a comparison of daily diaries and self-report questionnaires among low back pain patients. Primary Health Care Research & Development 2010;**11**:93-102.
15. Patrick DL, Deyo RA, Atlas SJ, Singer DE, Chapin AM, Keller RB. Assessing health-related quality of life in patients with sciatica. Spine 1995;**20**:1899-1908.

For peer review only

For peer review only - http://bmjopen.bmj.com/site/about/guidelines.xhtml

16. Dunn KM, Jordan K, Croft PR. Does questionnaire structure influence response in postal surveys? J Clin Epidemiol 2003;**56**:10-16.
17. Von Korff M, Ormel J, Keefe FJ, Dworkin SF. Grading the severity of chronic pain. Pain 1992;**50**:133-149.
18. Dunn KM, de Vet HCW, Hooper H, Ong BN, Croft PR. Measurement of back pain episode inception in questionnaires: a study combining quantitative and qualitative methods. Journal of Musculoskeletal Pain 2006;**14**:29-37.
19. Zigmond AS, Snaith RP. The Hospital Anxiety and Depression Scale. Acta Psychiatr Scand 1983;**67**:361-370.
20. Jenkins CD, Stanton BA, Niemcryk SJ, Rose RM. A scale for the estimation of sleep problems in clinical research. J Clin Epidemiol 1988;**41**:313-321.
21. Morphy H, Dunn KM, Lewis M, Boardman HF, Croft PR. Epidemiology of insomnia: a longitudinal study in a UK population. Sleep 2007;**30**:274-280.
22. Kroenke K, Spitzer RL, Williams JB. The PHQ-15: validity of a new measure for evaluating the severity of somatic symptoms. Psychosom Med 2002;**64**:258-266.
23. Waddell G. The Back Pain Revolution. Edinburgh: Churchill Livingstone; 1998.
24. Hestbaek L, Leboeuf-Yde C, Manniche C. Low back pain: what is the long-term course? A review of studies of general patient populations. Eur Spine J 2003;**12**:149-165.
25. Young AE, Wasiak R, Phillips L, Gross DP. Workers' perspectives on low back pain recurrence: "It comes and goes and comes and goes, but it's always there". Pain 2011;**152**:204-211.
26. Tamcan O, Mannion AF, Eisenring C, Horisberger B, Elfering A, Müller U. The course of chronic and recurrent low back pain in the general population. Pain 2010;**150**:451-457.
27. Raspe H. (2010) Measuring the impact of chronic pain on populations: a narrative review. In: Croft P, Blyth FM, van der Windt D, editors. Chronic pain epidemiology: From aetiology to public health. 1st ed. Oxford: Oxford University Press. pp. 61-70.
28. Hill JC, Vohora K, Dunn KM, Main CJ, Hay E. Comparing the STarT Back screening tool's subgroup allocation of individual patients with that of independent clinical experts. Clin J Pain 2010;**26**: 783-787.
29. Von Korff M, Dunn KM. Chronic pain reconsidered. Pain 2008;**138**:267-276.
30. Savigny P, Kuntze S, Watson P, Underwood M, Ritchie G, Cotterell M. (2009) Early management of persistent non-specific low back pain. London, National Collaborating Centre for Primary Care and Royal College of General Practitioners.
31. Chou R, Qaseem A, Snow V, Casey D, Cross JT, Jr., Shekelle P et al. Diagnosis and treatment of low back pain: a joint clinical practice guideline from the American College of Physicians and the American Pain Society. Ann Intern Med 2007;**147**:478-491.
32. Dunn KM, Jordan KP, Mancl L, Drangsholt MT, Le Resche L. Trajectories of pain in adolescents: a prospective cohort study. Pain 2011;**152**:66-73.

For peer review only

For peer review only - http://bmjopen.bmj.com/site/about/guidelines.xhtml

33. Aggarwal VR, McBeth J, Zakrzewska JM, Lunt M, Macfarlane GJ. The epidemiology of chronic syndromes that are frequently unexplained: do they have common associated factors? Int J Epidemiol 2006;**35**:468-476.

34. Nimnuan C, Rabe-Hesketh S, Wessely S, Hotopf M. How many functional somatic syndromes? J Psychosom Res 2001;**51**:549-557.

For peer review only

For peer review only - http://bmjopen.bmj.com/site/about/guidelines.xhtml

Appendix

Latent class analysis

The assumption behind the longitudinal latent class analysis was that there exists distinct pathways of back pain, and hence the participants can be grouped into distinct clusters (known as latent classes) based on their profile of back pain over the 6-months, with each subject belonging to one cluster. Specifically, longitudinal latent class analysis aims to obtain the smallest number of clusters that accounts for the associations between the monthly pain levels.

Latent class models were fitted successively, starting with a one cluster model and then sequentially adding another cluster for each successive model. There is no gold standard goodness of fit criteria for longitudinal latent class analysis models and so the final number of clusters was determined by examining the optimal models based on each of Akaike's Information criterion (AIC, and revised version AIC3), Bayes information criterion and the Consistent Akaike's Information criterion (1). The optimal number of clusters for each criterion is where the information criterion value is at its lowest. The percentage reduction in the model fit likelihood ratio chi-squared statistic (L^2) from the model with one cluster, was also calculated, with the optimal number of clusters where the percentage reduction is considered minor. The resultant optimal models were then compared on size (clusters should include at least 10% of participants) and with regards to having distinct cluster characteristics to determine the final number of clusters. LatentGold version 4.0 was used to perform the analyses. LatentGold uses both the EM and Newton-Raphson algorithms to estimate model parameters. 1000 different random starting values were used, each of which included 100 iterations. The bivariate residuals were used to assess violation of the local independence assumption for the optimal model. Local independence means that within clusters the

For peer review only - http://bmjopen.bmj.com/site/about/guidelines.xhtml

probability of a certain level of pain for any month is not related to the level of pain for any other month. Restricted latent class analysis models to address any violation were developed where the bivariate residuals between monthly pain ratings was greater than the recommended level of 1 (2).

The goodness of fit statistics for the longitudinal latent class analysis are shown in the Appendix Table 1. Between 3 and 6 clusters were considered optimal by the different goodness of fit measures, but the 6 cluster model included a cluster with only 2% of participants and so was dropped from consideration. A restricted 4 cluster model was ultimately selected as optimal, as the clusters were distinct, and included at least 10% of participants in each cluster. The fifth cluster in the 5-cluster model was a subgroup of a cluster in the 4-cluster model and did not have distinct characteristics.

An alternative to longitudinal latent class analysis which explicitly takes the time order into account is latent class growth analysis. Derivation of clusters using latent class growth analysis (not shown here) yielded similar trajectories presumably due to the relative stability of pain in participants. The pain profiles of individuals in each longitudinal latent class analysis cluster matched that of the cluster as a whole, and the clusters themselves revealed distinct pathways of pain and related health status. Some of the health status measures exhibited some skewness in scores but analysis comparing median and interquartile range scores showed the same patterns across clusters and led to the same conclusions as for the main analysis.

For peer review only - http://bmjopen.bmj.com/site/about/guidelines.xhtml

Appendix Table 1. Goodness of fit statistics for the longitudinal latent class analysis

Model	L^2	% reduction in L^2 from H_0	AIC_{LL}	$AIC3_{LL}$	BIC_{LL}	$CAIC_{LL}$
1 Cluster (H_0)	1150·56	-	1759·65	1773·65	1800·43	1814·43
2 Cluster	748·35	35	1373·44	1395·44	1437·52	1459·52
3 Cluster	556·74	52	1197·83	1227·83	1285·21[a]	1315·21[a]
4 Cluster	528·27	54	1185·36	1223·36	1296·04	1334·04
5 Cluster	501·80	56	1174·90	1220·90	1308·89	1354·88
6 Cluster	476·81	59	1165·90[a]	1219·90[a]	1323·19	1377·19
7 Cluster	461·89	60	1166·98	1228·98	1347·56	1409·56
8 Cluster	447·62	61	1168·71	1238·71	1372·60	1442·60

[a] optimal unrestricted model for that goodness of fit statistic

AIC= Akaike's Information criterion; BIC= Bayes information criterion; CAIC= Consistent Akaike's Information criterion.

For peer review only - http://bmjopen.bmj.com/site/about/guidelines.xhtml

For peer review only

Appendix References

(1) Ware JE, Kolinski M, Keller.S.D. How to score the SF-12 Physical and Mental Health Summaries: A User's Manual. Boston, MA: The Health Institute, New England Medical Centre; 1995.

(2) Magidson J, Vermunt JK. Latent Class Models. In: Kaplan DW, ed. The Sage Handbook of Quantitative Methodology for the Social Sciences. Thousand Oaks, CA: Sage Publications; 2004: 175-98

For peer review only

For peer review only - http://bmjopen.bmj.com/site/about/guidelines.xhtml

STROBE 2007 (v4) Statement—Checklist of items that should be included in reports of *cohort studies*

Section/Topic	Item #	Recommendation	Reported on page #
**Title and abstract**	1	(*a*) Indicate the study's design with a commonly used term in the title or the abstract	1
		(*b*) Provide in the abstract an informative and balanced summary of what was done and what was found	2
**Introduction**			
Background/rationale	2	Explain the scientific background and rationale for the investigation being reported	4
Objectives	3	State specific objectives, including any prespecified hypotheses	5
**Methods**			
Study design	4	Present key elements of study design early in the paper	5-7
Setting	5	Describe the setting, locations, and relevant dates, including periods of recruitment, exposure, follow-up, and data collection	5-6
Participants	6	(*a*) Give the eligibility criteria, and the sources and methods of selection of participants. Describe methods of follow-up	5-7
		(*b*) For matched studies, give matching criteria and number of exposed and unexposed	n/a
Variables	7	Clearly define all outcomes, exposures, predictors, potential confounders, and effect modifiers. Give diagnostic criteria, if applicable	6-7
Data sources/ measurement	8*	For each variable of interest, give sources of data and details of methods of assessment (measurement). Describe comparability of assessment methods if there is more than one group	*6-7*
Bias	9	Describe any efforts to address potential sources of bias	7-9
Study size	10	Explain how the study size was arrived at	5-6
Quantitative variables	11	Explain how quantitative variables were handled in the analyses. If applicable, describe which groupings were chosen and why	7-9
Statistical methods	12	(*a*) Describe all statistical methods, including those used to control for confounding	7-9
		(*b*) Describe any methods used to examine subgroups and interactions	7-9
		(*c*) Explain how missing data were addressed	7-9
		(*d*) If applicable, explain how loss to follow-up was addressed	7-9
		(*e*) Describe any sensitivity analyses	9
**Results**			

For peer review only

Participants	13*	(a) Report numbers of individuals at each stage of study—eg numbers potentially eligible, examined for eligibility, confirmed eligible, included in the study, completing follow-up, and analysed	9
		(b) Give reasons for non-participation at each stage	5-6 & 9 & 11
		(c) Consider use of a flow diagram	
Descriptive data	14*	(a) Give characteristics of study participants (eg demographic, clinical, social) and information on exposures and potential confounders	17-18
		(b) Indicate number of participants with missing data for each variable of interest	5-6 & 9 & 11
		(c) Summarise follow-up time (eg, average and total amount)	5-6
Outcome data	15*	Report numbers of outcome events or summary measures over time	9-10
Main results	16	(*a*) Give unadjusted estimates and, if applicable, confounder-adjusted estimates and their precision (eg, 95% confidence interval). Make clear which confounders were adjusted for and why they were included	n/a
		(*b*) Report category boundaries when continuous variables were categorized	6-7
		(*c*) If relevant, consider translating estimates of relative risk into absolute risk for a meaningful time period	n/a
Other analyses	17	Report other analyses done—eg analyses of subgroups and interactions, and sensitivity analyses	11
**Discussion**			
Key results	18	Summarise key results with reference to study objectives	12
**Limitations**			
Interpretation	20	Give a cautious overall interpretation of results considering objectives, limitations, multiplicity of analyses, results from similar studies, and other relevant evidence	12-14
Generalisability	21	Discuss the generalisability (external validity) of the study results	12
**Other information**			
Funding	22	Give the source of funding and the role of the funders for the present study and, if applicable, for the original study on which the present article is based	20

*Give information separately for cases and controls in case-control studies and, if applicable, for exposed and unexposed groups in cohort and cross-sectional studies.

Note: An Explanation and Elaboration article discusses each checklist item and gives methodological background and published examples of transparent reporting. The STROBE checklist is best used in conjunction with this article (freely available on the Web sites of PLoS Medicine at http://www.plosmedicine.org/, Annals of Internal Medicine at http://www.annals.org/, and Epidemiology at http://www.epidem.com/). Information on the STROBE Initiative is available at www.strobe-statement.org.

For peer review only

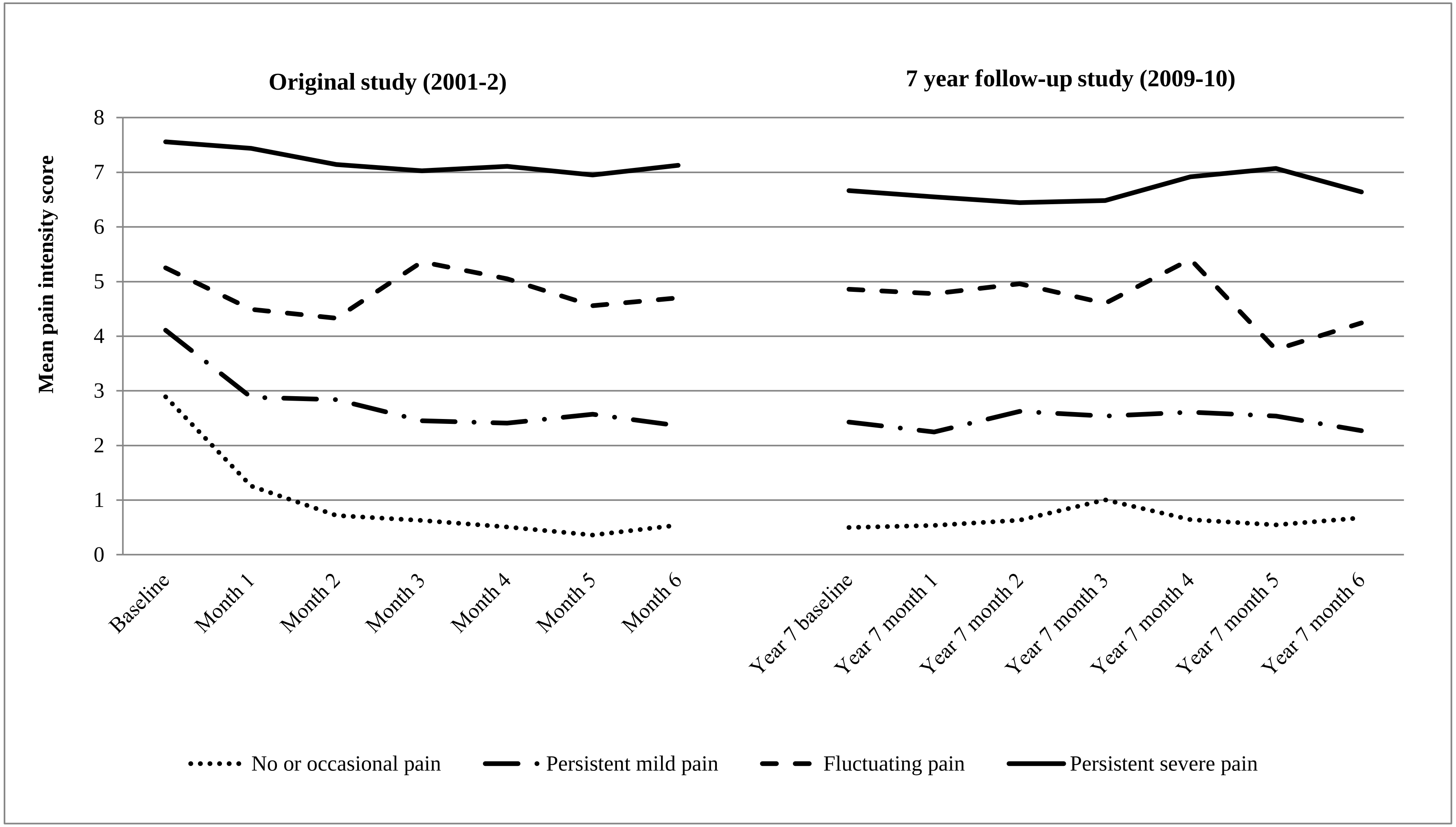
Original study (2001-2)
7 year follow-up study (2009-10)
Mean pain intensity score
8
7
6
5
4
3
2
1
0
Baseline
Month 1
Month 2
Month 3
Month 4
Month 5
Month 6
Year 7 baseline
Year 7 month 1
Year 7 month 2
Year 7 month 3
Year 7 month 4
Year 7 month 5
Year 7 month 6
No or occasional pain
Persistent mild pain
Fluctuating pain
Persistent severe pain

Long-term trajectories of back pain: cohort study with seven year follow-up

Journal:	*BMJ Open*
Manuscript ID:	bmjopen-2013-003838.R2
Article Type:	Research
Date Submitted by the Author:	08-Nov-2013
Complete List of Authors:	Dunn, Kate; Keele University, Arthritis Research UK Primary Care Centre<br>Campbell, Paul; Keele University, Arthritis Research UK Primary Care Centre<br>Jordan, Kelvin; Keele University, Arthritis Research UK Primary Care Centre
**Primary Subject Heading**:	Epidemiology
Secondary Subject Heading:	Rheumatology
Keywords:	EPIDEMIOLOGY, PRIMARY CARE, RHEUMATOLOGY

SCHOLARONE™ Manuscripts

For peer review only

Title: Long-term trajectories of back pain: cohort study with seven year follow-up

Authors: Kate M Dunn*, Paul Campbell*, Kelvin P Jordan*

Author affiliations

*Arthritis Research UK Primary Care Centre, Primary Care Sciences, Keele University, Staffordshire, ST5 5BG, UK.

Corresponding Authors Details

Dr Kate M Dunn, Arthritis Research UK Primary Care Centre, Primary Care Sciences, Keele University, Staffordshire, ST5 5BG, UK.

Tel: + 44 (0) 1782 734703; Fax: + 44 (0) 1782 733911, Email: k.m.dunn@keele.ac.uk

http://www.keele.ac.uk/research/pchs/pcmrc/

Keywords: Low Back Pain; Prospective Studies; Cluster Analysis; Longitudinal Studies; Pain Measurement.

Word count: 2793.

For peer review only - http://bmjopen.bmj.com/site/about/guidelines.xhtml

Abstract

Objective: To describe long-term trajectories of back pain.

Design: Monthly data collection for 6-months at 7-year follow-up of participants in a prospective cohort study.

Setting: Primary care practices in Staffordshire, UK.

Participants: 228 people consulting their GP with back pain, on whom information on 6-month back pain trajectories had been collected during 2001-3, and who had valid consent and contact details in 2009-10, were contacted. 155 participants (68% of those contacted) responded and provided sufficient data for primary analyses.

Outcome measures: Trajectories based on patients' self-reports of back pain, identified using Longitudinal Latent Class Analysis. Trajectories characterised using information on disability, psychological status and presence of other symptoms.

Results: Four clusters with different back pain trajectories at follow-up were identified: (i) no or occasional pain, (ii) persistent mild pain, (iii) fluctuating pain, and (iv) persistent severe pain. Trajectory clusters differed significantly from each other in terms of disability, psychological status and other symptoms. Most participants remained in a similar trajectory as seven years previously (weighted kappa 0.54; 95% CI 0.42, 0.65).

Conclusions: Most people with back pain appear to follow a particular pain trajectory over long time periods, and do not have frequently recurring or widely fluctuating patterns. Results are limited by lack of information about the time between data collection periods, and by loss to follow-up. However, findings do raise questions about standard divisions into acute and chronic back pain. A new framework for understanding the course of back pain is

For peer review only

proposed.

For peer review only

For peer review only - http://bmjopen.bmj.com/site/about/guidelines.xhtml

Article Summary

Article focus

- Most research studies have limited follow-up in terms of frequency of data collection and long-term timing
- Previous work has used frequent data collection points to identify new short-term trajectories of back pain
- This study aimed to carry out long-term follow-up of people in those trajectories to identify the long-term course and trajectories of back pain.

Key messages

- Four clusters with different back pain trajectories and characteristics at follow-up were identified: (i) no or occasional pain, (ii) persistent mild pain, (iii) fluctuating pain, and (iv) persistent severe pain.
- Most participants remained in a similar trajectory as seven years previously, indicating that people with back pain follow a particular pain trajectory over long time periods.
- Findings raise questions about standard divisions into acute and chronic back pain based purely on duration of current episode.

Strengths and limitations of this study

- The study benefits from long-term follow-up, prospective design, frequent follow-up during study periods, robust analyses and use of validated questionnaire instruments.
- The study was limited by loss to follow-up, meaning restricted numbers for full analysis, but multiple imputation was used to investigate the implications of this.
- Data collection phases were 7-years apart, and similar information about trajectories in the interim period is unavailable.

For peer review only

•

Introduction

Back pain is common – it has been recently highlighted as the single leading cause of years lived with disability worldwide (1) and many people experience pain over long periods. Among primary care consulters, 38% report having their symptoms for over three years.(2) Even among people in primary care with acute back pain, 75% report previous back pain,(3) indicating that even if not constantly present, back pain is a long-term experience. This has led to a suggestion to use a longer-term, lifecourse approach to studying back pain.(4)

The long-term experience of back pain is often not addressed by researchers. In a recent review of back pain prognosis, only 1 of the 33 included studies had follow-up beyond a year.(5) Studies with shorter term follow-up can only represent a compressed view of the long-term pain experience. The few longer-term studies have limited numbers of follow-up points,(6,7,8) Knowledge of prognosis is important, as stratifying back pain management based on risk of poor prognosis can be clinically and cost-effective,(9) with benefits for targeting early treatment and referrals. However previous research is unable to fully reflect the detailed course of back pain over time, or inform about long-term prognosis.

In 2001-2 we studied a cohort of people consulting in primary care with back pain.(10) We identified four distinct clusters of people with different trajectories: (i) recovering, (ii) persistent mild, (iii) fluctuating pain, (iv) severe chronic back pain. Duration of back pain at baseline increased with rising severity of trajectory, potentially indicating phases of increasing severity in the long-term course. This is supported by models of stages of back

For peer review only - http://bmjopen.bmj.com/site/about/guidelines.xhtml

pain chronicity (11) and degeneration with age.(12) Alternatively, trajectories could represent distinct groups with stable long-term pain. We aimed to describe long-term trajectories of back pain through a 6 month follow-up period of a cohort of back pain patients previously studied seven years earlier.

Methods

This is a follow-up of participants in a back pain cohort study whose short term (6 month) back pain trajectories had been derived in 2001-2.(10)

Study participants

The original study identified people aged 30-59 years consulting with back pain at one of five general practices in North Staffordshire, UK, during 2001-2. Details are published elsewhere.(13) Briefly, participants returning baseline questionnaires and consenting to follow-up were sent monthly questionnaires. Those returning four or more questionnaires during the first six months were included in a longitudinal latent class analysis to determine trajectories of back pain.(10) Of the 342 participants in this original analysis, 73% (n=250) gave their consent to be contacted again. In 2009, current contact details were not available for 22 (6%), leaving 228 people from the original analysis invited to take part at seven year follow-up.

For peer review only - http://bmjopen.bmj.com/site/about/guidelines.xhtml

Data collection at seven years

Self-completion questionnaires were mailed to the 228 study participants (seven year baseline mailing) with reminders at two and four weeks, and brief questionnaires for non-responders at six weeks. Participants giving informed consent were sent brief monthly questionnaires for six months (the same data collection technique as the original study).

All questionnaires contained the same key measures. Pain intensity was measured using the mean of three 0-10 numerical rating scales.(14) Disability was measured using the modified 23-item Roland-Morris Disability Questionnaire (RMDQ).(15) These instruments were used in the original study,(10) and there is evidence of reliability in UK primary care back pain patients.(16) The Chronic Pain Grade classified individuals into grades of chronic pain;(17) this was included in the brief seven year baseline mailing for non-responders. Back pain duration was recalled time since the last pain-free month.(18)

The Hospital Anxiety and Depression Scale (HADS) was used to assess psychological status.(19) It produces scores from 0-21, with higher scores indicating more severe symptoms. Insomnia was defined as reporting having trouble falling or staying asleep, waking up several times a night, or waking up feeling tired on most nights.(20) This definition has been used previously in pain samples.(21) Somatic symptoms were measured using the 15-item Patient Health Questionnaire (PHQ-15) (22) which is scored from 0 (not bothered with any symptoms) to 30 (bothered a lot with 15 symptoms). Leg pain was self-

For peer review only - http://bmjopen.bmj.com/site/about/guidelines.xhtml

reported pain travelling from the back to the leg(s), and upper body pain was self-reported pain in the shoulder, arm, neck or head, during the previous two weeks.

Analysis

For peer review only

Two primary analysis groups were formed from responders to this seven-year follow-up study. Group one participants returned the seven year baseline questionnaire plus three or more questionnaires from months one to six. Group two included participants with insufficient seven year follow-up data for full analyses, but who provided adequate information for multiple imputation to be carried out.

For Group one participants, monthly back pain intensity scores were trichotomized into no pain (scoring less than one), mild-moderate pain, and high pain (scored five or more). Longitudinal latent class analysis was used to group participants into clusters based on the trajectory of their back pain over these six months as in the original study.(10) In longitudinal latent class analysis, each participant is allocated to the cluster best matching their pain profile, based on each participant's probability of belonging to each cluster, with participants allocated to the cluster for which they have the largest probability. Participants should be clearly assigned to a single cluster with high probability. Cluster-specific probabilities of having each level of pain for each month, given membership of that cluster, allow development of pain pathways for each cluster. See appendix for more details.

For peer review only - http://bmjopen.bmj.com/site/about/guidelines.xhtml

For Group two participants, the multiple imputation procedure in Stata/IC v11.1 software with 50 imputations, through a multinomial logistic regression, was used to impute membership of the seven year clusters identified for Group one. Information on cluster from the original study, plus outcome measures from the seven year baseline questionnaire were used to impute cluster membership.

Membership of clusters from both study phases (original and seven year follow-up) were compared to investigate long-term patterns of trajectory membership. Stability of cluster membership was assessed using weighted kappa. Kappa can be interpreted as agreement (stability) between original and seven year follow-up cluster memberships beyond chance, with values of 1 indicating perfect agreement and 0 indicating agreement no better than chance. The seven year derived clusters (actual or imputed) were compared on the key measures of the seven year baseline questionnaire, using simple linear or logistic regression as appropriate through the multiple imputation estimate commands in Stata/IC v11.1.

In order to address potential issues from loss to follow-up from the original 2001-3 trajectories analysis, an additional Group three was formed. This included everyone from the original analysis who was not included in the primary analysis at 7 years (above): seven-year responders who provided insufficient data, non-responders at seven years, people who could not be traced, and those not giving consent to follow-up. Groups one and two combined were compared to Group three on baseline demographic, pain, anxiety and depression from the original study using t-tests or chi-squared tests as appropriate.

For peer review only - http://bmjopen.bmj.com/site/about/guidelines.xhtml

As sensitivity analysis, seven year cluster membership was imputed for Group two and Group three participants using information from the original study (baseline Roland-Morris Disability Questionnaire, Chronic Pain Grade, pain duration and original longitudinal latent class analysis cluster). Comparisons between the original cluster and seven year actual or imputed cluster membership for participants across all three groups were performed.

For peer review only

Ethics Statement

The original study and the 2009-10 follow-up phase were independently approved by North Staffordshire Local Research Ethics Committee and South Staffordshire Research Ethics Committee respectively.

Results

Primary analyses were carried out on 155 responders (68% of the 228 contacted): 112 in Group one (full data available) and 43 in Group two (imputation required).

Clusters at seven year follow-up

The optimal number of clusters resulting from longitudinal latent class analysis was four (see appendix). 84% of Group one participants had an average probability of greater than 0.90 of

For peer review only - http://bmjopen.bmj.com/site/about/guidelines.xhtml

being allocated to their assigned cluster, indicating distinct classification. Group two participants were allocated to these clusters using multiple imputation.

The estimated probability of monthly levels of pain within clusters is shown in Table 1. These monthly probabilities of pain can be interpreted to describe the occurrence of pain, for example, a probability of mild-moderate pain of 0.13 at baseline for the first Cluster indicates that one in every eight people in that group are likely to have experienced mild-moderate pain that month. The first cluster identified (31% of Group one and Group two) mostly had no pain (estimated monthly probabilities of no pain 0.65-0.87), with occasional mild episodes (cluster labelled 'no or occasional pain'). Participants in this cluster generally reported no pain on at least 4 occasions over the six months and did not report high pain. The second cluster (37%) had mild pain intensity most of the time, with a maximum of 1-2 months of no pain; only 17% of the cluster ever reported high pain. Their monthly probabilities of mild pain were between 0.69-0.91 ('persistent mild pain'). The third cluster (11%) had pain fluctuating between mild and high levels ('fluctuating pain'), and rarely reported no pain. The final cluster (21%) had high pain intensity levels throughout, with monthly probabilities of high pain between 0.79-0.98 ('persistent severe pain'), and never reported no pain.

Comparison of clusters from original study and seven year follow-up

The identified trajectories of back pain intensity for the original study and the seven year follow-up are illustrated in Figure 1.

For peer review only - http://bmjopen.bmj.com/site/about/guidelines.xhtml

Most participants stayed in a similar cluster between the two study phases (weighted kappa 0.54 (95% confidence interval (CI) 0.42, 0.65)) (Table 2). 74% (95% CI 57%, 92%) of those originally in the most severe trajectory remained in an equivalent cluster at seven years. Over half the participants in the two mildest clusters in the original study (recovering: 59%; 95% CI 44%, 74%; persistent mild pain: 56%; 95% CI 40%, 73%) stayed in the most comparable trajectory at seven years, and most who changed moved to the other mild trajectory. The fluctuating group in the original study (the smallest group) did not show a stable pattern, with 87% of participants changing cluster, mainly to persistent mild or persistent severe clusters.

Pain intensity, disability and psychological status all differed significantly between the seven year trajectories, with the no or occasional pain cluster having the lowest disability levels (mean RMDQ score 2.0), least pain intensity (mean 0.8) and best psychological status (mean HADS depression score 2.8), and the persistent severe pain cluster having the highest disability (mean RMDQ score 12.9), worst pain intensity (mean 6.7) and poorest psychological status (mean HADS depression score 7.4) (Table 3). Similar statistically significant differences were also present in the original study.(10) The clusters also differed significantly in terms of the presence of somatic symptoms and insomnia, with the mean symptom score (PHQ-15) ranging from 3.9 in the no or occasional pain group to 7.7 in the persistent severe pain cluster, and the proportion classified with insomnia ranging from 27% to 80%.

Sensitivity analyses

For peer review only - http://bmjopen.bmj.com/site/about/guidelines.xhtml

For peer review only

Group three comprised 25 seven-year responders who provided insufficient data, 48 non-responders at seven years, plus the people from the original study who did not give consent to follow-up (n=92) or could not be traced (n=22). Original study baseline characteristics of the three Groups are shown in Table 4. The only significant difference between participants in Groups one and two and those in Group three was gender, with fewer females in Group three (p=0.04).

Including imputed data from Group three participants as well as Group two made little difference to the estimated relative sizes of the seven year clusters reported above, and gave similar patterns of disability, psychological status and other symptoms.

For peer review only

Discussion

This study provides unique prospective data on the long-term course of back pain. It suggests that most people remain in a particular pain trajectory, with similar characteristics, when estimated in two periods at the beginning and end of a seven year period. These findings do not support the hypotheses that there are phases, or degeneration, in the course of back pain over time. Our findings show that widely fluctuating pain is not common (the fluctuating cluster was consistently smallest), and most people have pain patterns varying slightly around their own mean long-term pain. This includes people who recover quickly, and maintain very low (or no) pain, and people who have persistently higher levels of pain. Descriptions of back pain often assume a prevailing pattern of recurrent or fluctuating pain.(23;24) Our findings, and recent qualitative work,(25) provide evidence that these opinions do not give the full picture. However, our study reports pain trajectories among individuals who have sought healthcare, and although recent work identifying general population trajectories of back pain showed trajectories similar to ours,(26) their fluctuating cluster comprised more of the population (35%).

Strengths of the current study include the long-term nature, prospective design, frequent follow-up during study periods, robust analyses and use of validated questionnaire instruments. However, the study did suffer from loss to follow-up, meaning limited numbers for full analysis. Multiple imputation was used to investigate the implications of this, and participants included in primary analyses were similar to those excluded, but the possibility

For peer review only - http://bmjopen.bmj.com/site/about/guidelines.xhtml

For peer review only

of selection bias and residual confounding cannot be ruled out. Although this study had frequent follow-up points during data collection phases, these phases were 7-years apart, and information about trajectories in the interim period is unavailable and therefore unknown.

Few studies have suggested models for long-term change in back pain. Our study gives some support to the model by Raspe et al,(11) as worsening back pain trajectory was significantly associated with more disability, distress, other pains and symptoms, similar to their model of symptom ‘amplification’. However, the prospective nature of our study indicates that this ‘amplification’ is not related to deterioration over time or stages of change, but describes underlying differences between groups of people whose general pattern of pain does not appear to change over time. In addition, it appears that the spread of pain, further complaints and depressive symptoms increases fairly consistently with increasing severity of pain trajectory, rather than occurring in discrete stages, as in the amplification model.(11;27) Our results also do not support models of degeneration with age,(12) as clusters do not differ by age. Our findings suggest a new framework model for the long-term course of back pain, comprising four different types of back pain trajectory, each with characteristic pain patterns, disability levels, psychological status and wider symptoms.

New research is emerging on the treatment of back pain according to prognostic risk groups,(9) but questions have been raised about timing of risk group allocation.(28) Our research highlights potentially stable groups of people with different pain trajectories and characteristics. Comparison of the two study phases showed that no cluster changed mean Roland-Morris Disability Questionnaire score by over 2.5 points (a recommended clinically important change for back pain). This knowledge could improve allocation of treatment

For peer review only - http://bmjopen.bmj.com/site/about/guidelines.xhtml

according to prognostic risk. However, collecting data over six months to allocate treatment is not clinically plausible, and work is needed to identify pain trajectories concisely and accurately. An important implication of our findings is that classifying back pain simply as acute or chronic is insufficient. This is apparent when standard chronic pain definitions would group people with persistent mild symptoms with people who experience constant high levels of pain and other symptoms. Previous work has also highlighted problems defining acute and chronic pain,(25;29) but clinical guidelines are still formulated on this basis.(30;31) Researchers and clinicians should begin to rely less on standard definitions of back pain.

This study raises questions of when, during the life course, trajectory membership is determined. Adolescent trajectories of back pain showed some similar features to the current study (e.g. a cluster with very high probability of pain), whereas other trajectories indicated development of a pain condition.(32) Comparable trajectories were also identified for headache, facial pain and stomach pain in the adolescent cohort,(32) which indicates potential applicability of these findings to other conditions, particularly non-specific symptoms.(33;34)

Conclusions

We have provided unique evidence on the long-term course of back pain, and suggested a new framework for understanding the course of the condition. There is evidence against phases of change in back pain over time. There are limitations of the study, such as the lack of information about the time between data collection periods, but if the results apply to a significant proportion of back pain patients, there are important clinical implications. First, a large proportion of those who do report initial pain recover quickly, but among those who do

For peer review only - http://bmjopen.bmj.com/site/about/guidelines.xhtml

not, our results show that many will remain in the same trajectory when assessed several years later. Second, if people in the most severe trajectories could be identified when seeking healthcare, they could be directed to specific targeted treatments. The current study provides substantial new understanding of the long-term course of back pain, and has the potential to have impact in both research and clinical arenas.

For peer review only

For peer review only - http://bmjopen.bmj.com/site/about/guidelines.xhtml

Table 1. Monthly probability of experiencing each level of back pain based on cluster membership at 7-years

For peer review only

For peer review only - http://bmjopen.bmj.com/site/about/guidelines.xhtml

	Cluster (trajectory) from 7-year follow-up analysis			
	No / occasional pain	Persistent mild pain	Fluctuating pain	Persistent severe pain
Baseline				
No pain	0·87	0·15	0·01	0·00
Mild-moderate pain	0·13	0·80	0·51	0·21
High pain	0·00	0·05	0·48	0·79
Month 1				
No pain	0·85	0·06	0·00	0·00
Mild-moderate pain	0·15	0·91	0·62	0·17
High pain	0·00	0·04	0·38	0·83
Month 2				
No pain	0·65	0·06	0·00	0·00
Mild-moderate pain	0·35	0·89	0·12	0·11
High pain	0·00	0·05	0·88	0·89
Month 3				
No pain	0·70	0·07	0·01	0·00
Mild-moderate pain	0·30	0·86	0·58	0·17
High pain	0·00	0·07	0·42	0·83
Month 4				
No pain	0·66	0·09	0·00	0·00
Mild-moderate pain	0·34	0·88	0·29	0·18
High pain	0·00	0·03	0·71	0·82
Month 5				
No pain	0·75	0·26	0·19	0·00
Mild-moderate pain	0·25	0·69	0·73	0·02
High pain	0·00	0·05	0·09	0·98
Month 6				
No pain	0·80	0·16	0·02	0·00
Mild-moderate pain	0·20	0·79	0·66	0·11
High pain	0·00	0·05	0·32	0·89

For peer review only

For peer review only - http://bmjopen.bmj.com/site/about/guidelines.xhtml

Table 2. Cluster membership at 7 years stratified by original study cluster (n=155)

Original study cluster	No. in original study cluster	*n* (%)[a] in each cluster (trajectory) at 7 years			
		No or occasional pain	Persistent mild pain	Fluctuating pain	Persistent severe pain
Recovering	57	34 (59%)	18 (32%)	3 (5%)	2 (4%)
Persistent mild	51	12 (23%)	29 (56%)	8 (15%)	2 (5%)
Fluctuating	16	1 (7%)	6 (38%)	2 (13%)	7 (42%)
Severe chronic	31	0 (0%)	4 (12%)	4 (14%)	23 (74%)

[a] estimated following multiple imputation

Weighted kappa = 0.54 (95% CI 0.42, 0.65)

For peer review only - http://bmjopen.bmj.com/site/about/guidelines.xhtml

Table 3. Characteristics of cluster membership at 7-year baseline follow-up, Group 1 and 2 (n=155)

	Cluster (trajectory) from 7-year follow-up analysis				
	No or occasional pain	Persistent mild pain	Fluctuating pain	Persistent severe pain	p-value
% in cluster	31%	37%	11%	21%	
Age	46·3 (43·9, 48·6)	47·7 (45·5, 50·0)	46·3 (42·1, 50·6)	47·0 (43·7, 50·2)	0·85
Female	65% (51, 80)	63% (50, 77)	68% (43, 93)	63% (45, 81)	0·99
Pain intensity	0·8 (0, 1·8)	2·3 (1·8, 2·8)	4·9 (3·6, 6·3)	6·7 (5·8, 7·6)	<0·001
Leg pain	42% (26, 58)	51% (37, 65)	78% (54, 100)	83% (68, 98)	0·009
Upper body pain	52% (36, 68)	71% (58, 84)	88% (71, 100)	93% (84, 100)	0·004
Disability	2·0 (0, 4·1)	4·3 (3·0, 5·6)	8·7 (5·7, 11·7)	12·9 (10·5, 15·3)	<0·001
Anxiety	5·3 (4·1, 6·4)	6·8 (5·6, 8·0)	6·5 (4·4, 8·6)	8·8 (7·3, 10·3)	0·005
Depression	2·8 (1·8, 3·8)	4·9 (3·8, 6·0)	4·3 (2·8, 5·8)	7·4 (5·9, 8·8)	<0·001
PHQ 15	3·9 (2·6, 5·3)	5·0 (3·9, 6·1)	7·4 (4·3, 10·4)	7·7 (5·8, 9·7)	0·006
Insomnia	27% (12, 42)	42% (28, 57)	75% (51, 98)	80% (65, 96)	<0·001

Figures are mean (95% confidence interval) except female, leg pain, upper body pain and insomnia, which are percentage (95% confidence interval). PHQ-15 = Patient Health Questionnaire.

For peer review only - http://bmjopen.bmj.com/site/about/guidelines.xhtml

Table 4. Original study baseline characteristics of study participants

	Full 7-year follow-up (Group 1: n=112)	Limited 7-year follow-up (Group 2: n=43)	Groups 1 & 2: (n=155)	No 7-year follow-up data available (Group 3: n=187)	*p*-value: Groups 1&2 v. Group 3
Gender (female)†	72 (64%)	28 (65%)	100 (65%)	100 (53%)	0·04
Age (years)	46·9 (8·3)	47·0 (7·7)	47·0 (8·1)	47·4 (8·2)	0·63
Pain intensity	4·4 (2·7)	4·5 (2·9)	4·4 (2·8)	4·7 (2·5)	0·26
Disability	9·1 (6·8)	10·7 (6·8)	9·5 (6·8)	10·6 (6·4)	0·14
CPG IV†	30 (28%)	17 (40%)	47 (31%)	57 (32%)	0·86
Anxiety	8·2 (4.8)	9·1 (4·6)	8·5 (4·8)	8·6 (4·9)	0·82
Depression	6·1 (4·4)	8·4 (4·9)	6·8 (4·6)	7·5 (4·8)	0·15
Duration of pain†					
<= 6 months	42 (38%)	14 (33%)	56 (36%)	51 (28%)	0·10
7-35 months	23 (21%)	14 (33%)	37 (24%)	48 (26%)	
>= 3 years	46 (41%)	15 (35%)	61 (40%)	85 (46%)	
Cluster†					
Recovering	42 (38%)	15 (35%)	57 (37%)	47 (25%)	0·10
Persistent mild	34 (30%)	17 (40%)	51 (33%)	71 (38%)	
Fluctuating	13 (12%)	3 (7%)	16 (10%)	29 (16%)	
Severe-chronic	23 (21%)	8 (19%)	31 (20%)	40 (21%)	

Figures are mean (standard deviation) except those marked † which are numbers (percentage). CPG IV = Chronic Pain Grade IV

For peer review only - http://bmjopen.bmj.com/site/about/guidelines.xhtml

Figure 1. Trajectories of back pain intensity from original study and 7-year follow-up

For peer review only

For peer review only - http://bmjopen.bmj.com/site/about/guidelines.xhtml

Acknowledgements

We thank all the participants and general practices who participated in the original and follow-up phase of this study. We thank Professor Peter Croft for his comments on drafts of the paper.

Competing Interest statement

The authors have declared that no competing interests exist.

Funding statement

The work was supported by the Wellcome Trust grant number 083572. The funding body had no role in study design, data collection and analysis, decision to publish, or preparation of the manuscript.

Author's contributions

Kate Dunn conceived the study. All authors contributed to the design of the study. Paul Campbell and Kate Dunn coordinated the data collection. Kate Dunn and Kelvin Jordan analysed the data. All authors interpreted the data. Kate Dunn drafted the manuscript and all authors contributed to revisions. Kate Dunn had full access to all of the data in the study and takes responsibility for the integrity of the data and the accuracy of the data analysis. All authors approved the final version of the manuscript submitted for publication.

For peer review only

Checklist

STROBE statement enclosed.

Data sharing statement

The Arthritis Research UK Primary Care Centre has established data sharing arrangements to support joint publications and other research collaborations. Applications for access to anonymised data from our research databases are reviewed by the Centre's Data Custodian and Academic Proposal (DCAP) Committee and a decision regarding access to the data is made subject to the NRES ethical approval first provided for the study and to new analysis being proposed. Further information on our data sharing procedures can be found on the Centre's website (http://www.keele.ac.uk/pchs/publications/datasharingresources/) or by emailing the Centre's data manager (primarycare.datasharing@keele.ac.uk).

For peer review only

For peer review only - http://bmjopen.bmj.com/site/about/guidelines.xhtml

References

1. Vos T, Flaxman AD, Naghavi M, et al. Years lived with disability (YLDs) for 1160 sequelae of 289 diseases and injuries 1990—2010: a systematic analysis for the Global Burden of Disease Study 2010. The Lancet 2012;**380**: 2163 – 2196.
2. Dunn KM, Croft PR. The importance of symptom duration in determining prognosis. Pain 2006;**121**:126-132.
3. Henschke N, Maher CG, Refshauge KM, et al. Prognosis in patients with recent onset low back pain in Australian primary care: inception cohort study. BMJ 2008;**337**:a171.
4. Dunn KM. Extending conceptual frameworks: life course epidemiology for the study of back pain. BMC Musculoskelet Disord 2010;**11**:23.
5. Costa LD, Maher CG, Hancock MJ, et al. The prognosis of acute and persistent low-back pain: a meta-analysis. Canadian Medical Association Journal 2012;**84**:E613-E624.
6. Enthoven P, Skargren E, Carstensen J, et al. Predictive factors for 1-year and 5-year outcome for disability in a working population of patients with low back pain treated in primary care. Pain 2006;**122**:137-144.
7. Burton AK, McClune TD, Clarke RD, et al. Long-term follow-up of patients with low back pain attending for manipulative care-outcomes and predictors. Man Ther 2004;**9**:30-35.
8. Von Korff M, Miglioretti DL. A prognostic approach to defining chronic pain. Pain 2005;**117**:304-313.
9. Hill JC, Whitehurst DG, Lewis M, et al. Comparison of stratified primary care management for low back pain with current best practice (STarT Back): a randomised controlled trial. The Lancet 2011;**378**: 1560-1571.
10. Dunn KM, Jordan K, Croft PR. Characterising the course of low back pain: a latent class analysis. Am J Epidemiol 2006;**163**:754-761.
11. Raspe H, Huppe A, Matthis C. Theories and models of chronicity: on the way to a broader definition of chronic back pain. Schmerz 2003;**17**:359-366.
12. Laurens Rowe M. Backache at work. Fairport, New York: Perinton Press; 1983.
13. Dunn KM, Croft PR. Classification of low back pain in primary care: using "bothersomeness" to identify the most severe cases. Spine 2005;**30**:1887-1892.
14. Dunn KM, Jordan KP, Croft PR. Recall of medication use, self-care activities and pain intensity: a comparison of daily diaries and self-report questionnaires among low back pain patients. Primary Health Care Research & Development 2010;**11**:93-102.
15. Patrick DL, Deyo RA, Atlas SJ, et al. Assessing health-related quality of life in patients with sciatica. Spine 1995;**20**:1899-1908.
16. Dunn KM, Jordan K, Croft PR. Does questionnaire structure influence response in postal surveys? J Clin Epidemiol 2003;**56**:10-16.

For peer review only - http://bmjopen.bmj.com/site/about/guidelines.xhtml

17. Von Korff M, Ormel J, Keefe FJ, et al. Grading the severity of chronic pain. Pain 1992;**50**:133-149.

18. Dunn KM, de Vet HCW, Hooper H, et al. Measurement of back pain episode inception in questionnaires: a study combining quantitative and qualitative methods. Journal of Musculoskeletal Pain 2006;**14**:29-37.

19. Zigmond AS, Snaith RP. The Hospital Anxiety and Depression Scale. Acta Psychiatr Scand 1983;**67**:361-370.

20. Jenkins CD, Stanton BA, Niemcryk SJ, et al. A scale for the estimation of sleep problems in clinical research. J Clin Epidemiol 1988;**41**:313-321.

21. Morphy H, Dunn KM, Lewis M, et al. Epidemiology of insomnia: a longitudinal study in a UK population. Sleep 2007;**30**:274-280.

22. Kroenke K, Spitzer RL, Williams JB. The PHQ-15: validity of a new measure for evaluating the severity of somatic symptoms. Psychosom Med 2002;**64**:258-266.

23. Waddell G. The Back Pain Revolution. Edinburgh: Churchill Livingstone; 1998.

24. Hestbaek L, Leboeuf-Yde C, Manniche C. Low back pain: what is the long-term course? A review of studies of general patient populations. Eur Spine J 2003;**12**:149-165.

25. Young AE, Wasiak R, Phillips L, et al. Workers' perspectives on low back pain recurrence: "It comes and goes and comes and goes, but it's always there". Pain 2011;**152**:204-211.

26. Tamcan O, Mannion AF, Eisenring C, et al. The course of chronic and recurrent low back pain in the general population. Pain 2010;**150**:451-457.

27. Raspe H. (2010) Measuring the impact of chronic pain on populations: a narrative review. In: Croft P, Blyth FM, van der Windt D, editors. Chronic pain epidemiology: From aetiology to public health. 1st ed. Oxford: Oxford University Press. pp. 61-70.

28. Hill JC, Vohora K, Dunn KM, et al. Comparing the STarT Back screening tool's subgroup allocation of individual patients with that of independent clinical experts. Clin J Pain 2010;**26**: 783-787.

29. Von Korff M, Dunn KM. Chronic pain reconsidered. Pain 2008;**138**:267-276.

30. Savigny P, Kuntze S, Watson P, et al. (2009) Early management of persistent non-specific low back pain. London, National Collaborating Centre for Primary Care and Royal College of General Practitioners.

31. Chou R, Qaseem A, Snow V, et al. Diagnosis and treatment of low back pain: a joint clinical practice guideline from the American College of Physicians and the American Pain Society. Ann Intern Med 2007;**147**:478-491.

32. Dunn KM, Jordan KP, Mancl L, et al. Trajectories of pain in adolescents: a prospective cohort study. Pain 2011;**152**:66-73.

For peer review only - http://bmjopen.bmj.com/site/about/guidelines.xhtml

33. Aggarwal VR, McBeth J, Zakrzewska JM, et al. The epidemiology of chronic syndromes that are frequently unexplained: do they have common associated factors? Int J Epidemiol 2006;**35**:468-476.

34. Nimnuan C, Rabe-Hesketh S, Wessely S, et al. How many functional somatic syndromes? J Psychosom Res 2001;**51**:549-557.

For peer review only

For peer review only - http://bmjopen.bmj.com/site/about/guidelines.xhtml

For peer review only

For peer review only - http://bmjopen.bmj.com/site/about/guidelines.xhtml

Title: Long-term trajectories of back pain: cohort study with seven year follow-up

Authors: Kate M Dunn*, Paul Campbell*, Kelvin P Jordan*

Author affiliations

*Arthritis Research UK Primary Care Centre, Primary Care Sciences, Keele University, Staffordshire, ST5 5BG, UK.

Corresponding Authors Details

Dr Kate M Dunn, Arthritis Research UK Primary Care Centre, Primary Care Sciences, Keele University, Staffordshire, ST5 5BG, UK.

Tel: + 44 (0) 1782 734703; Fax: + 44 (0) 1782 733911, Email: k.m.dunn@keele.ac.uk

http://www.keele.ac.uk/research/pchs/pcmrc/

Keywords: Low Back Pain; Prospective Studies; Cluster Analysis; Longitudinal Studies; Pain Measurement.

Word count: ~~2767~~2793.

For peer review only

For peer review only - http://bmjopen.bmj.com/site/about/guidelines.xhtml

Abstract

Objective: To describe long-term trajectories of back pain.

Design: Monthly data collection for 6-months at 7-year follow-up of participants in a prospective cohort study.

Setting: Primary care practices in Staffordshire, UK.

Participants: 228 people consulting their GP with back pain, on whom information on 6-month back pain trajectories had been collected during 2001-3, and who had valid consent and contact details in 2009-10, were contacted. 155 participants (68% of those contacted) responded and provided sufficient data for primary analyses.

Outcome measures: Trajectories based on patients' self-reports of back pain, identified using Longitudinal Latent Class Analysis. Trajectories characterised using information on disability, psychological status and presence of other symptoms.

Results: Four clusters with different back pain trajectories at follow-up were identified: (i) no or occasional pain, (ii) persistent mild pain, (iii) fluctuating pain, and (iv) persistent severe pain. Trajectory clusters differed significantly from each other in terms of disability, psychological status and other symptoms. Most participants remained in a similar trajectory as seven years previously (weighted kappa 0.54; 95% CI 0.42, 0.65).

Conclusions: Most people with back pain appear to follow a particular pain trajectory over long time periods, and do not have frequently recurring or widely fluctuating patterns. Results are limited by lack of information about the time between data collection periods, and by loss to follow-up. However, findings do raise questions about standard divisions into acute and chronic back pain. A new framework for understanding the course of back pain is proposed.

For peer review only

Article Summary

Article focus

- Most research studies have limited follow-up in terms of frequency of data collection and long-term timing
- Previous work has used frequent data collection points to identify new short-term trajectories of back pain
- This study aimed to carry out long-term follow-up of people in those trajectories to identify the long-term course and trajectories of back pain.

Key messages

- Four clusters with different back pain trajectories and characteristics at follow-up were identified: (i) no or occasional pain, (ii) persistent mild pain, (iii) fluctuating pain, and (iv) persistent severe pain.
- Most participants remained in a similar trajectory as seven years previously, indicating that people with back pain follow a particular pain trajectory over long time periods.
- Findings raise questions about standard divisions into acute and chronic back pain based purely on duration of current episode.

Strengths and limitations of this study

- The study benefits from long-term follow-up, prospective design, frequent follow-up during study periods, robust analyses and use of validated questionnaire instruments.
- The study was limited by loss to follow-up, meaning restricted numbers for full analysis, but multiple imputation was used to investigate the implications of this.
- Data collection phases were 7-years apart, and similar information about trajectories in the interim period is unavailable.

For peer review only

Introduction

Back pain is common – it has been recently highlighted as the single leading cause of years lived with disability worldwide (1) and many people experience pain over long periods. Among primary care consulters, 38% report having their symptoms for over three years.(2) Even among people in primary care with acute back pain, 75% report previous back pain,(3) indicating that even if not constantly present, back pain is a long-term experience. This has led to a suggestion to use a longer-term, lifecourse approach to studying back pain.(4)

The long-term experience of back pain is often not addressed by researchers. In a recent review of back pain prognosis, only 1 of the 33 included studies had follow-up beyond a year.(5) Studies with shorter term follow-up can only represent a compressed view of the long-term pain experience. The few longer-term studies have limited numbers of follow-up points,(6,7,8) Knowledge of prognosis is important, as stratifying back pain management based on risk of poor prognosis can be clinically and cost-effective,(9) with benefits for targeting early treatment and referrals. However previous research is unable to fully reflect the detailed course of back pain over time, or inform about long-term prognosis.

In 2001-2 we studied a cohort of people consulting in primary care with back pain.(10) We identified four distinct clusters of people with different trajectories: (i) recovering, (ii) persistent mild, (iii) fluctuating pain, (iv) severe chronic back pain. Duration of back pain at baseline increased with rising severity of trajectory, potentially indicating phases of increasing severity in the long-term course. This is supported by models of stages of back

For peer review only - http://bmjopen.bmj.com/site/about/guidelines.xhtml

pain chronicity (11) and degeneration with age.(12) Alternatively, trajectories could represent distinct groups with stable long-term pain. We aimed to describe long-term trajectories of back pain through a 6 month follow-up period of a cohort of back pain patients previously studied seven years earlier.

Methods

This is a follow-up of participants in a back pain cohort study whose short term (6 month) back pain trajectories had been derived in 2001-2.(10)

Study participants

The original study identified people aged 30-59 years consulting with back pain at one of five general practices in North Staffordshire, UK, during 2001-2. Details are published elsewhere.(13) Briefly, participants returning baseline questionnaires and consenting to follow-up were sent monthly questionnaires. Those returning four or more questionnaires during the first six months were included in a longitudinal latent class analysis to determine trajectories of back pain.(10) Of the 342 participants in this original analysis, 73% (n=250) gave their consent to be contacted again. In 2009, current contact details were not available for 22 (6%), leaving 228 people from the original analysis invited to take part at seven year follow-up.

For peer review only - http://bmjopen.bmj.com/site/about/guidelines.xhtml

Data collection at seven years

Self-completion questionnaires were mailed to the 228 study participants (seven year baseline mailing) with reminders at two and four weeks, and brief questionnaires for non-responders at six weeks. Participants giving informed consent were sent brief monthly questionnaires for six months (the same data collection technique as the original study).

All questionnaires contained the same key measures. Pain intensity was measured using the mean of three 0-10 numerical rating scales.(14) Disability was measured using the modified 23-item Roland-Morris Disability Questionnaire (RMDQ).(15) These instruments were used in the original study,(10) and there is evidence of reliability in UK primary care back pain patients.(16) The Chronic Pain Grade classified individuals into grades of chronic pain;(17) this was included in the brief seven year baseline mailing for non-responders. Back pain duration was recalled time since the last pain-free month.(18)

The Hospital Anxiety and Depression Scale (HADS) was used to assess psychological status.(19) It produces scores from 0-21, with higher scores indicating more severe symptoms. Insomnia was defined as reporting having trouble falling or staying asleep, waking up several times a night, or waking up feeling tired on most nights.(20) This definition has been used previously in pain samples.(21) Somatic symptoms were measured using the 15-item Patient Health Questionnaire (PHQ-15) (22) which is scored from 0 (not bothered with any symptoms) to 30 (bothered a lot with 15 symptoms). Leg pain was self-

For peer review only - http://bmjopen.bmj.com/site/about/guidelines.xhtml

reported pain travelling from the back to the leg(s), and upper body pain was self-reported pain in the shoulder, arm, neck or head, during the previous two weeks.

Analysis

Two primary analysis groups were formed from responders to this seven-year follow-up study. Group one participants returned the seven year baseline questionnaire plus three or more questionnaires from months one to six. Group two included participants with insufficient seven year follow-up data for full analyses, but who provided adequate information for multiple imputation to be carried out.

For Group one participants, monthly back pain intensity scores were trichotomized into no pain (scoring less than one), mild-moderate pain, and high pain (scored five or more). Longitudinal latent class analysis was used to group participants into clusters based on the trajectory of their back pain over these six months as in the original study.(10) In longitudinal latent class analysis, each participant is allocated to the cluster best matching their pain profile, based on each participant's probability of belonging to each cluster, with participants allocated to the cluster for which they have the largest probability. Participants should be clearly assigned to a single cluster with high probability. Cluster-specific probabilities of having each level of pain for each month, given membership of that cluster, allow development of pain pathways for each cluster. See appendix for more details.

For peer review only - http://bmjopen.bmj.com/site/about/guidelines.xhtml

For Group two participants, the multiple imputation procedure in Stata/IC v11.1 software with 50 imputations, through a multinomial logistic regression, was used to impute membership of the seven year clusters identified for Group one. Information on cluster from the original study, plus outcome measures from the seven year baseline questionnaire were used to impute cluster membership.

Membership of clusters from both study phases (original and seven year follow-up) were compared to investigate long-term patterns of trajectory membership. Stability of cluster membership was assessed using weighted kappa. Kappa can be interpreted as agreement (stability) between original and seven year follow-up cluster memberships beyond chance, with values of 1 indicating perfect agreement and 0 indicating agreement no better than chance. The seven year derived clusters (actual or imputed) were compared on the key measures of the seven year baseline questionnaire, using simple linear or logistic regression as appropriate through the multiple imputation estimate commands in Stata/IC v11.1.

In order to address potential issues from loss to follow-up from the original 2001-3 trajectories analysis, an additional Group three was formed. This included everyone from the original analysis who was not included in the primary analysis at 7 years (above): seven-year responders who provided insufficient data, non-responders at seven years, people who could not be traced, and those not giving consent to follow-up. Groups one and two combined were compared to Group three on baseline demographic, pain, anxiety and depression from the original study using t-tests or chi-squared tests as appropriate.

For peer review only - http://bmjopen.bmj.com/site/about/guidelines.xhtml

As sensitivity analysis, seven year cluster membership was imputed for Group two and Group three participants using information from the original study (baseline Roland-Morris Disability Questionnaire, Chronic Pain Grade, pain duration and original longitudinal latent class analysis cluster). Comparisons between the original cluster and seven year actual or imputed cluster membership for participants across all three groups were performed.

Ethics Statement

The original study and the 2009-10 follow-up phase were independently approved by North Staffordshire Local Research Ethics Committee and South Staffordshire Research Ethics Committee respectively.

Results

Primary analyses were carried out on 155 responders (68% of the 228 contacted): 112 in Group one (full data available) and 43 in Group two (imputation required).

Clusters at seven year follow-up

The optimal number of clusters resulting from longitudinal latent class analysis was four (see appendix). 84% of Group one participants had an average probability of greater than 0.90 of

For peer review only - http://bmjopen.bmj.com/site/about/guidelines.xhtml

being allocated to their assigned cluster, indicating distinct classification. Group two participants were allocated to these clusters using multiple imputation.

The estimated probability of monthly levels of pain within clusters is shown in Table 1. These monthly probabilities of pain can be interpreted to describe the occurrence of pain, for example, a probability of mild-moderate pain of 0.13 at baseline for the first Cluster indicates that one in every eight people in that group are likely to have experienced mild-moderate pain that month. The first cluster identified (31% of Group one and Group two) mostly had no pain (estimated monthly probabilities of no pain 0.65-0.87), with occasional mild episodes (cluster labelled 'no or occasional pain'). Participants in this cluster generally reported no pain on at least 4 occasions over the six months and did not report high pain. The second cluster (37%) had mild pain intensity most of the time, with a maximum of 1-2 months of no pain; only 17% of the cluster ever reported high pain. Their monthly probabilities of mild pain were between 0.69-0.91 ('persistent mild pain'). The third cluster (11%) had pain fluctuating between mild and high levels ('fluctuating pain'), and rarely reported no pain. The final cluster (21%) had high pain intensity levels throughout, with monthly probabilities of high pain between 0.79-0.98 ('persistent severe pain'), and never reported no pain.

Comparison of clusters from original study and seven year follow-up

The identified trajectories of back pain intensity for the original study and the seven year follow-up are illustrated in Figure 1.

For peer review only - http://bmjopen.bmj.com/site/about/guidelines.xhtml

Most participants stayed in a similar cluster between the two study phases (weighted kappa 0.54 (95% confidence interval (CI) 0.42, 0.65)) (Table 2). 74% (95% CI 57%, 92%) of those originally in the most severe trajectory remained in an equivalent cluster at seven years. Over half the participants in the two mildest clusters in the original study (recovering: 59%; 95% CI 44%, 74%; persistent mild pain: 56%; 95% CI 40%, 73%) stayed in the most comparable trajectory at seven years, and most who changed moved to the other mild trajectory. The fluctuating group in the original study (the smallest group) did not show a stable pattern, with 87% of participants changing cluster, mainly to persistent mild or persistent severe clusters.

Pain intensity, disability and psychological status all differed significantly between the seven year trajectories, with the no or occasional pain cluster having the lowest disability levels (mean RMDQ score 2.0), least pain intensity (mean 0.8) and best psychological status (mean HADS depression score 2.8), and the persistent severe pain cluster having the highest disability (mean RMDQ score 12.9), worst pain intensity (mean 6.7) and poorest psychological status (mean HADS depression score 7.4) (Table 3). Similar statistically significant differences were also present in the original study.(10) The clusters also differed significantly in terms of the presence of somatic symptoms and insomnia, with the mean symptom score (PHQ-15) ranging from 3.9 in the no or occasional pain group to 7.7 in the persistent severe pain cluster, and the proportion classified with insomnia ranging from 27% to 80%.

Sensitivity analyses

For peer review only - http://bmjopen.bmj.com/site/about/guidelines.xhtml

Group three comprised 25 seven-year responders who provided insufficient data, 48 non-responders at seven years, plus the people from the original study who did not give consent to follow-up (n=92) or could not be traced (n=22). Original study baseline characteristics of the three Groups are shown in Table 4. The only significant difference between participants in Groups one and two and those in Group three was gender, with fewer females in Group three (p=0.04).

Including imputed data from Group three participants as well as Group two made little difference to the estimated relative sizes of the seven year clusters reported above, and gave similar patterns of disability, psychological status and other symptoms.

For peer review only

For peer review only - http://bmjopen.bmj.com/site/about/guidelines.xhtml

Discussion

This study provides unique prospective data on the long-term course of back pain. It suggests that most people remain in a particular pain trajectory, with similar characteristics, when estimated ~~across~~ in two periods at the beginning and end of a seven year period. These findings do not support the hypotheses that there are phases, or degeneration, in the course of back pain over time. Our findings show that widely fluctuating pain is not common (the fluctuating cluster was consistently smallest), and most people have pain patterns varying slightly around their own mean long-term pain. This includes people who recover quickly, and maintain very low (or no) pain, and people who have persistently higher levels of pain. Descriptions of back pain often assume a prevailing pattern of recurrent or fluctuating pain.(23;24) Our findings, and recent qualitative work,(25) provide evidence that these opinions do not give the full picture. However, our study reports pain trajectories among individuals who have sought healthcare, and although recent work identifying general population trajectories of back pain showed trajectories similar to ours,(26) their fluctuating cluster comprised more of the population (35%).

Strengths of the current study include the long-term nature, prospective design, frequent follow-up during study periods, robust analyses and use of validated questionnaire instruments. However, the study did suffer from loss to follow-up, meaning limited numbers for full analysis. Multiple imputation was used to investigate the implications of this, and participants included in primary analyses were similar to those excluded, but the possibility of selection bias and residual confounding cannot be ruled out. Although this study had frequent follow-up points during data collection phases, these~~, data collection~~ phases were 7-

For peer review only - http://bmjopen.bmj.com/site/about/guidelines.xhtml

years apart, and similar information about trajectories in the interim period is unavailable and therefore unknown.

Few studies have suggested models for long-term change in back pain. Our study gives some support to the model by Raspe et al,(11) as worsening back pain trajectory was significantly associated with more disability, distress, other pains and symptoms, similar to their model of symptom 'amplification'. However, the prospective nature of our study indicates that this 'amplification' is not related to deterioration over time or stages of change, but describes underlying differences between groups of people whose general pattern of pain does not appear to change over time. In addition, it appears that the spread of pain, further complaints and depressive symptoms increases fairly consistently with increasing severity of pain trajectory, rather than occurring in discrete stages, as in the amplification model.(11;27) Our results also do not support models of degeneration with age,(12) as clusters do not differ by age. Our findings suggest a new framework model for the long-term course of back pain, comprising four different types of back pain trajectory, each with characteristic pain patterns, disability levels, psychological status and wider symptoms.

New research is emerging on the treatment of back pain according to prognostic risk groups,(9) but questions have been raised about timing of risk group allocation.(28) Our research highlights potentially stable groups of people with different pain trajectories and characteristics. Comparison of the two study phases showed that no cluster changed mean Roland-Morris Disability Questionnaire score by over 2.5 points (a recommended clinically important change for back pain). This knowledge could improve allocation of treatment according to prognostic risk. However, collecting data over six months to allocate treatment

For peer review only - http://bmjopen.bmj.com/site/about/guidelines.xhtml

is not clinically plausible, and work is needed to identify pain trajectories concisely and accurately. An important implication of our findings is that classifying back pain simply as acute or chronic is insufficient. This is apparent when standard chronic pain definitions would group people with persistent mild symptoms with people who experience constant high levels of pain and other symptoms. Previous work has also highlighted problems defining acute and chronic pain,(25;29) but clinical guidelines are still formulated on this basis.(30;31) Researchers and clinicians should begin to rely less on standard definitions of back pain.

This study raises questions of when, during the life course, trajectory membership is determined. Adolescent trajectories of back pain showed some similar features to the current study (e.g. a cluster with very high probability of pain), whereas other trajectories indicated development of a pain condition.(32) Comparable trajectories were also identified for headache, facial pain and stomach pain in the adolescent cohort,(32) which indicates potential applicability of these findings to other conditions, particularly non-specific symptoms.(33;34)

Conclusions

We have provided unique evidence on the long-term course of back pain, and suggested a new framework for understanding the course of the condition. There is evidence against phases of change in back pain over time. There are ~~some potential~~ limitations of the study, such as the lack of information about the time between data collection periods, but~~,~~ if the results apply to a significant proportion of back pain patients, there are important clinical implications. First, a large proportion of those who do report initial pain recover quickly, but among those who do not, our results show that many will remain in the same trajectory ~~over~~

For peer review only - http://bmjopen.bmj.com/site/about/guidelines.xhtml

~~the longer-term~~when assessed several years later. Second, if people in the most severe trajectories could be identified when seeking healthcare, they could be directed to specific targeted treatments. The current study provides substantial new understanding of the long-term course of back pain, and has the potential to have impact in both research and clinical arenas.

For peer review only

For peer review only - http://bmjopen.bmj.com/site/about/guidelines.xhtml

Table 1. Monthly probability of experiencing each level of back pain based on cluster membership at 7-years

	Cluster (trajectory) from 7-year follow-up analysis			
	No / occasional pain	Persistent mild pain	Fluctuating pain	Persistent severe pain
Baseline				
No pain	0·87	0·15	0·01	0·00
Mild-moderate pain	0·13	0·80	0·51	0·21
High pain	0·00	0·05	0·48	0·79
Month 1				
No pain	0·85	0·06	0·00	0·00
Mild-moderate pain	0·15	0·91	0·62	0·17
High pain	0·00	0·04	0·38	0·83
Month 2				
No pain	0·65	0·06	0·00	0·00
Mild-moderate pain	0·35	0·89	0·12	0·11
High pain	0·00	0·05	0·88	0·89
Month 3				
No pain	0·70	0·07	0·01	0·00
Mild-moderate pain	0·30	0·86	0·58	0·17
High pain	0·00	0·07	0·42	0·83
Month 4				
No pain	0·66	0·09	0·00	0·00
Mild-moderate pain	0·34	0·88	0·29	0·18
High pain	0·00	0·03	0·71	0·82
Month 5				
No pain	0·75	0·26	0·19	0·00
Mild-moderate pain	0·25	0·69	0·73	0·02
High pain	0·00	0·05	0·09	0·98
Month 6				
No pain	0·80	0·16	0·02	0·00
Mild-moderate pain	0·20	0·79	0·66	0·11
High pain	0·00	0·05	0·32	0·89

For peer review only - http://bmjopen.bmj.com/site/about/guidelines.xhtml

Table 2. Cluster membership at 7 years stratified by original study cluster (n=155)

Original study cluster	No. in original study cluster	*n* (%)[a] in each cluster (trajectory) at 7 years			
		No or occasional pain	Persistent mild pain	Fluctuating pain	Persistent severe pain
Recovering	57	34 (59%)	18 (32%)	3 (5%)	2 (4%)
Persistent mild	51	12 (23%)	29 (56%)	8 (15%)	2 (5%)
Fluctuating	16	1 (7%)	6 (38%)	2 (13%)	7 (42%)
Severe chronic	31	0 (0%)	4 (12%)	4 (14%)	23 (74%)

[a] estimated following multiple imputation

Weighted kappa = 0.54 (95% CI 0.42, 0.65)

For peer review only - http://bmjopen.bmj.com/site/about/guidelines.xhtml

Table 3. Characteristics of cluster membership at 7-year baseline follow-up, Group 1 and 2 (n=155)

	Cluster (trajectory) from 7-year follow-up analysis				
	No or occasional pain	Persistent mild pain	Fluctuating pain	Persistent severe pain	p-value
% in cluster	31%	37%	11%	21%	
Age	46·3 (43·9, 48·6)	47·7 (45·5, 50·0)	46·3 (42·1, 50·6)	47·0 (43·7, 50·2)	0·85
Female	65% (51, 80)	63% (50, 77)	68% (43, 93)	63% (45, 81)	0·99
Pain intensity	0·8 (0, 1·8)	2·3 (1·8, 2·8)	4·9 (3·6, 6·3)	6·7 (5·8, 7·6)	<0·001
Leg pain	42% (26, 58)	51% (37, 65)	78% (54, 100)	83% (68, 98)	0·009
Upper body pain	52% (36, 68)	71% (58, 84)	88% (71, 100)	93% (84, 100)	0·004
Disability	2·0 (0, 4·1)	4·3 (3·0, 5·6)	8·7 (5·7, 11·7)	12·9 (10·5, 15·3)	<0·001
Anxiety	5·3 (4·1, 6·4)	6·8 (5·6, 8·0)	6·5 (4·4, 8·6)	8·8 (7·3, 10·3)	0·005
Depression	2·8 (1·8, 3·8)	4·9 (3·8, 6·0)	4·3 (2·8, 5·8)	7·4 (5·9, 8·8)	<0·001
PHQ 15	3·9 (2·6, 5·3)	5·0 (3·9, 6·1)	7·4 (4·3, 10·4)	7·7 (5·8, 9·7)	0·006
Insomnia	27% (12, 42)	42% (28, 57)	75% (51, 98)	80% (65, 96)	<0·001

Figures are mean (95% confidence interval) except female, leg pain, upper body pain and insomnia, which are percentage (95% confidence interval). PHQ-15 = Patient Health Questionnaire.

For peer review only

For peer review only - http://bmjopen.bmj.com/site/about/guidelines.xhtml

Table 4. Original study baseline characteristics of study participants

	Full 7-year follow-up (Group 1: n=112)	Limited 7-year follow-up (Group 2: n=43)	Groups 1 & 2: (n=155)	No 7-year follow-up data available (Group 3: n=187)	*p*-value: Groups 1&2 v. Group 3
Gender (female)†	72 (64%)	28 (65%)	100 (65%)	100 (53%)	0·04
Age (years)	46·9 (8·3)	47·0 (7·7)	47·0 (8·1)	47·4 (8·2)	0·63
Pain intensity	4·4 (2·7)	4·5 (2·9)	4·4 (2·8)	4·7 (2·5)	0·26
Disability	9·1 (6·8)	10·7 (6·8)	9·5 (6·8)	10·6 (6·4)	0·14
CPG IV†	30 (28%)	17 (40%)	47 (31%)	57 (32%)	0·86
Anxiety	8·2 (4.8)	9·1 (4·6)	8·5 (4·8)	8·6 (4·9)	0·82
Depression	6·1 (4·4)	8·4 (4·9)	6·8 (4·6)	7·5 (4·8)	0·15
Duration of pain†					
<= 6 months	42 (38%)	14 (33%)	56 (36%)	51 (28%)	0·10
7-35 months	23 (21%)	14 (33%)	37 (24%)	48 (26%)	
>= 3 years	46 (41%)	15 (35%)	61 (40%)	85 (46%)	
Cluster†					
Recovering	42 (38%)	15 (35%)	57 (37%)	47 (25%)	0·10
Persistent mild	34 (30%)	17 (40%)	51 (33%)	71 (38%)	
Fluctuating	13 (12%)	3 (7%)	16 (10%)	29 (16%)	
Severe-chronic	23 (21%)	8 (19%)	31 (20%)	40 (21%)	

Figures are mean (standard deviation) except those marked † which are numbers (percentage). CPG IV = Chronic Pain Grade IV

For peer review only - http://bmjopen.bmj.com/site/about/guidelines.xhtml

Figure 1. Trajectories of back pain intensity from original study and 7-year follow-up

For peer review only

For peer review only - http://bmjopen.bmj.com/site/about/guidelines.xhtml

Acknowledgements

We thank all the participants and general practices who participated in the original and follow-up phase of this study. We thank Professor Peter Croft for his comments on drafts of the paper.

Competing Interest statement

The authors have declared that no competing interests exist.

Funding statement

The work was supported by the Wellcome Trust grant number 083572. The funding body had no role in study design, data collection and analysis, decision to publish, or preparation of the manuscript.

Author's contributions

Kate Dunn conceived the study. All authors contributed to the design of the study. Paul Campbell and Kate Dunn coordinated the data collection. Kate Dunn and Kelvin Jordan analysed the data. All authors interpreted the data. Kate Dunn drafted the manuscript and all authors contributed to revisions. Kate Dunn had full access to all of the data in the study and takes responsibility for the integrity of the data and the accuracy of the data analysis. All authors approved the final version of the manuscript submitted for publication.

For peer review only

Checklist

STROBE statement enclosed.

Data sharing statement

The Arthritis Research UK Primary Care Centre has established data sharing arrangements to support joint publications and other research collaborations. Applications for access to anonymised data from our research databases are reviewed by the Centre's Data Custodian and Academic Proposal (DCAP) Committee and a decision regarding access to the data is made subject to the NRES ethical approval first provided for the study and to new analysis being proposed. Further information on our data sharing procedures can be found on the Centre's website (http://www.keele.ac.uk/pchs/publications/datasharingresources/) or by emailing the Centre's data manager (primarycare.datasharing@keele.ac.uk).

For peer review only

References

1. Vos T, Flaxman AD, Naghavi M, Lozano R, Michaud C et al. Years lived with disability (YLDs) for 1160 sequelae of 289 diseases and injuries 1990—2010: a systematic analysis for the Global Burden of Disease Study 2010. The Lancet 2012;**380**: 2163 – 2196.
2. Dunn KM, Croft PR. The importance of symptom duration in determining prognosis. Pain 2006;**121**:126-132.
3. Henschke N, Maher CG, Refshauge KM, Herbert RD, Cumming RG, Bleasel J et al. Prognosis in patients with recent onset low back pain in Australian primary care: inception cohort study. BMJ 2008;**337**:a171.
4. Dunn KM. Extending conceptual frameworks: life course epidemiology for the study of back pain. BMC Musculoskelet Disord 2010;**11**:23.
5. Costa LD, Maher CG, Hancock MJ, McAuley JH, Herbert RD, Costa LO. The prognosis of acute and persistent low-back pain: a meta-analysis. Canadian Medical Association Journal 2012;**84**:E613-E624.
6. Enthoven P, Skargren E, Carstensen J, Oberg B. Predictive factors for 1-year and 5-year outcome for disability in a working population of patients with low back pain treated in primary care. Pain 2006;**122**:137-144.
7. Burton AK, McClune TD, Clarke RD, Main CJ. Long-term follow-up of patients with low back pain attending for manipulative care-outcomes and predictors. Man Ther 2004;**9**:30-35.
8. Von Korff M, Miglioretti DL. A prognostic approach to defining chronic pain. Pain 2005;**117**:304-313.
9. Hill JC, Whitehurst DG, Lewis M, Bryan S, Dunn KM, Foster NE et al. Comparison of stratified primary care management for low back pain with current best practice (STarT Back): a randomised controlled trial. The Lancet 2011;**378**: 1560-1571.
10. Dunn KM, Jordan K, Croft PR. Characterising the course of low back pain: a latent class analysis. Am J Epidemiol 2006;**163**:754-761.
11. Raspe H, Huppe A, Matthis C. Theories and models of chronicity: on the way to a broader definition of chronic back pain. Schmerz 2003;**17**:359-366.
12. Laurens Rowe M. Backache at work. Fairport, New York: Perinton Press; 1983.
13. Dunn KM, Croft PR. Classification of low back pain in primary care: using "bothersomeness" to identify the most severe cases. Spine 2005;**30**:1887-1892.
14. Dunn KM, Jordan KP, Croft PR. Recall of medication use, self-care activities and pain intensity: a comparison of daily diaries and self-report questionnaires among low back pain patients. Primary Health Care Research & Development 2010;**11**:93-102.
15. Patrick DL, Deyo RA, Atlas SJ, Singer DE, Chapin AM, Keller RB. Assessing health-related quality of life in patients with sciatica. Spine 1995;**20**:1899-1908.

For peer review only

16. Dunn KM, Jordan K, Croft PR. Does questionnaire structure influence response in postal surveys? J Clin Epidemiol 2003;**56**:10-16.
17. Von Korff M, Ormel J, Keefe FJ, Dworkin SF. Grading the severity of chronic pain. Pain 1992;**50**:133-149.
18. Dunn KM, de Vet HCW, Hooper H, Ong BN, Croft PR. Measurement of back pain episode inception in questionnaires: a study combining quantitative and qualitative methods. Journal of Musculoskeletal Pain 2006;**14**:29-37.
19. Zigmond AS, Snaith RP. The Hospital Anxiety and Depression Scale. Acta Psychiatr Scand 1983;**67**:361-370.
20. Jenkins CD, Stanton BA, Niemcryk SJ, Rose RM. A scale for the estimation of sleep problems in clinical research. J Clin Epidemiol 1988;**41**:313-321.
21. Morphy H, Dunn KM, Lewis M, Boardman HF, Croft PR. Epidemiology of insomnia: a longitudinal study in a UK population. Sleep 2007;**30**:274-280.
22. Kroenke K, Spitzer RL, Williams JB. The PHQ-15: validity of a new measure for evaluating the severity of somatic symptoms. Psychosom Med 2002;**64**:258-266.
23. Waddell G. The Back Pain Revolution. Edinburgh: Churchill Livingstone; 1998.
24. Hestbaek L, Leboeuf-Yde C, Manniche C. Low back pain: what is the long-term course? A review of studies of general patient populations. Eur Spine J 2003;**12**:149-165.
25. Young AE, Wasiak R, Phillips L, Gross DP. Workers' perspectives on low back pain recurrence: "It comes and goes and comes and goes, but it's always there". Pain 2011;**152**:204-211.
26. Tamcan O, Mannion AF, Eisenring C, Horisberger B, Elfering A, Müller U. The course of chronic and recurrent low back pain in the general population. Pain 2010;**150**:451-457.
27. Raspe H. (2010) Measuring the impact of chronic pain on populations: a narrative review. In: Croft P, Blyth FM, van der Windt D, editors. Chronic pain epidemiology: From aetiology to public health. 1st ed. Oxford: Oxford University Press. pp. 61-70.
28. Hill JC, Vohora K, Dunn KM, Main CJ, Hay E. Comparing the STarT Back screening tool's subgroup allocation of individual patients with that of independent clinical experts. Clin J Pain 2010;**26**: 783-787.
29. Von Korff M, Dunn KM. Chronic pain reconsidered. Pain 2008;**138**:267-276.
30. Savigny P, Kuntze S, Watson P, Underwood M, Ritchie G, Cotterell M. (2009) Early management of persistent non-specific low back pain. London, National Collaborating Centre for Primary Care and Royal College of General Practitioners.
31. Chou R, Qaseem A, Snow V, Casey D, Cross JT, Jr., Shekelle P et al. Diagnosis and treatment of low back pain: a joint clinical practice guideline from the American College of Physicians and the American Pain Society. Ann Intern Med 2007;**147**:478-491.
32. Dunn KM, Jordan KP, Mancl L, Drangsholt MT, Le Resche L. Trajectories of pain in adolescents: a prospective cohort study. Pain 2011;**152**:66-73.

For peer review only - http://bmjopen.bmj.com/site/about/guidelines.xhtml

33. Aggarwal VR, McBeth J, Zakrzewska JM, Lunt M, Macfarlane GJ. The epidemiology of chronic syndromes that are frequently unexplained: do they have common associated factors? Int J Epidemiol 2006;**35**:468-476.

34. Nimnuan C, Rabe-Hesketh S, Wessely S, Hotopf M. How many functional somatic syndromes? J Psychosom Res 2001;**51**:549-557.

For peer review only

For peer review only - http://bmjopen.bmj.com/site/about/guidelines.xhtml

Appendix

Latent class analysis

The assumption behind the longitudinal latent class analysis was that there exists distinct pathways of back pain, and hence the participants can be grouped into distinct clusters (known as latent classes) based on their profile of back pain over the 6-months, with each subject belonging to one cluster. Specifically, longitudinal latent class analysis aims to obtain the smallest number of clusters that accounts for the associations between the monthly pain levels.

Latent class models were fitted successively, starting with a one cluster model and then sequentially adding another cluster for each successive model. There is no gold standard goodness of fit criteria for longitudinal latent class analysis models and so the final number of clusters was determined by examining the optimal models based on each of Akaike's Information criterion (AIC, and revised version AIC3), Bayes information criterion and the Consistent Akaike's Information criterion (1). The optimal number of clusters for each criterion is where the information criterion value is at its lowest. The percentage reduction in the model fit likelihood ratio chi-squared statistic (L^2) from the model with one cluster, was also calculated, with the optimal number of clusters where the percentage reduction is considered minor. The resultant optimal models were then compared on size (clusters should include at least 10% of participants) and with regards to having distinct cluster characteristics to determine the final number of clusters. LatentGold version 4.0 was used to perform the analyses. LatentGold uses both the EM and Newton-Raphson algorithms to estimate model parameters. 1000 different random starting values were used, each of which included 100 iterations. The bivariate residuals were used to assess violation of the local independence assumption for the optimal model. Local independence means that within clusters the

For peer review only - http://bmjopen.bmj.com/site/about/guidelines.xhtml

probability of a certain level of pain for any month is not related to the level of pain for any other month. Restricted latent class analysis models to address any violation were developed where the bivariate residuals between monthly pain ratings was greater than the recommended level of 1 (2).

The goodness of fit statistics for the longitudinal latent class analysis are shown in the Appendix Table 1. Between 3 and 6 clusters were considered optimal by the different goodness of fit measures, but the 6 cluster model included a cluster with only 2% of participants and so was dropped from consideration. A restricted 4 cluster model was ultimately selected as optimal, as the clusters were distinct, and included at least 10% of participants in each cluster. The fifth cluster in the 5-cluster model was a subgroup of a cluster in the 4-cluster model and did not have distinct characteristics.

An alternative to longitudinal latent class analysis which explicitly takes the time order into account is latent class growth analysis. Derivation of clusters using latent class growth analysis (not shown here) yielded similar trajectories presumably due to the relative stability of pain in participants. The pain profiles of individuals in each longitudinal latent class analysis cluster matched that of the cluster as a whole, and the clusters themselves revealed distinct pathways of pain and related health status. Some of the health status measures exhibited some skewness in scores but analysis comparing median and interquartile range scores showed the same patterns across clusters and led to the same conclusions as for the main analysis.

For peer review only - http://bmjopen.bmj.com/site/about/guidelines.xhtml

Appendix Table 1. Goodness of fit statistics for the longitudinal latent class analysis

Model	L^2	% reduction in L^2 from H_0	AIC_{LL}	$AIC3_{LL}$	BIC_{LL}	$CAIC_{LL}$
1 Cluster (H_0)	1150·56	-	1759·65	1773·65	1800·43	1814·43
2 Cluster	748·35	35	1373·44	1395·44	1437·52	1459·52
3 Cluster	556·74	52	1197·83	1227·83	1285·21[a]	1315·21[a]
4 Cluster	528·27	54	1185·36	1223·36	1296·04	1334·04
5 Cluster	501·80	56	1174·90	1220·90	1308·89	1354·88
6 Cluster	476·81	59	1165·90[a]	1219·90[a]	1323·19	1377·19
7 Cluster	461·89	60	1166·98	1228·98	1347·56	1409·56
8 Cluster	447·62	61	1168·71	1238·71	1372·60	1442·60

[a] optimal unrestricted model for that goodness of fit statistic

AIC= Akaike's Information criterion; BIC= Bayes information criterion; CAIC= Consistent Akaike's Information criterion.

For peer review only - http://bmjopen.bmj.com/site/about/guidelines.xhtml

Appendix References

(1) Ware JE, Kolinski M, Keller.S.D. How to score the SF-12 Physical and Mental Health Summaries: A User's Manual. Boston, MA: The Health Institute, New England Medical Centre; 1995.

(2) Magidson J, Vermunt JK. Latent Class Models. In: Kaplan DW, ed. The Sage Handbook of Quantitative Methodology for the Social Sciences. Thousand Oaks, CA: Sage Publications; 2004: 175-98

For peer review only

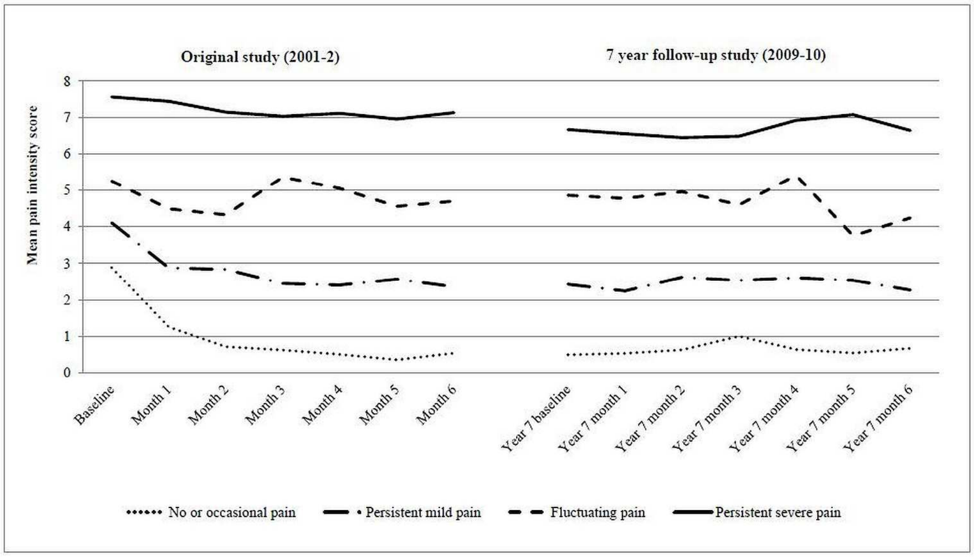

263x152mm (300 x 300 DPI)

review only

STROBE 2007 (v4) Statement—Checklist of items that should be included in reports of *cohort studies*

Section/Topic	Item #	Recommendation	Reported on page #
**Title and abstract**	1	(*a*) Indicate the study's design with a commonly used term in the title or the abstract	1
		(*b*) Provide in the abstract an informative and balanced summary of what was done and what was found	2
**Introduction**			
Background/rationale	2	Explain the scientific background and rationale for the investigation being reported	4
Objectives	3	State specific objectives, including any prespecified hypotheses	5
**Methods**			
Study design	4	Present key elements of study design early in the paper	5-7
Setting	5	Describe the setting, locations, and relevant dates, including periods of recruitment, exposure, follow-up, and data collection	5-6
Participants	6	(*a*) Give the eligibility criteria, and the sources and methods of selection of participants. Describe methods of follow-up	5-7
		(*b*) For matched studies, give matching criteria and number of exposed and unexposed	n/a
Variables	7	Clearly define all outcomes, exposures, predictors, potential confounders, and effect modifiers. Give diagnostic criteria, if applicable	6-7
Data sources/ measurement	8*	For each variable of interest, give sources of data and details of methods of assessment (measurement). Describe comparability of assessment methods if there is more than one group	*6-7*
Bias	9	Describe any efforts to address potential sources of bias	7-9
Study size	10	Explain how the study size was arrived at	5-6
Quantitative variables	11	Explain how quantitative variables were handled in the analyses. If applicable, describe which groupings were chosen and why	7-9
Statistical methods	12	(*a*) Describe all statistical methods, including those used to control for confounding	7-9
		(*b*) Describe any methods used to examine subgroups and interactions	7-9
		(*c*) Explain how missing data were addressed	7-9
		(*d*) If applicable, explain how loss to follow-up was addressed	7-9
		(*e*) Describe any sensitivity analyses	9
**Results**			

For peer review only

Participants	13*	(a) Report numbers of individuals at each stage of study—eg numbers potentially eligible, examined for eligibility, confirmed eligible, included in the study, completing follow-up, and analysed	9
		(b) Give reasons for non-participation at each stage	5-6 & 9 & 11
		(c) Consider use of a flow diagram	
Descriptive data	14*	(a) Give characteristics of study participants (eg demographic, clinical, social) and information on exposures and potential confounders	17-18
		(b) Indicate number of participants with missing data for each variable of interest	5-6 & 9 & 11
		(c) Summarise follow-up time (eg, average and total amount)	5-6
Outcome data	15*	Report numbers of outcome events or summary measures over time	9-10
Main results	16	(*a*) Give unadjusted estimates and, if applicable, confounder-adjusted estimates and their precision (eg, 95% confidence interval). Make clear which confounders were adjusted for and why they were included	n/a
		(*b*) Report category boundaries when continuous variables were categorized	6-7
		(*c*) If relevant, consider translating estimates of relative risk into absolute risk for a meaningful time period	n/a
Other analyses	17	Report other analyses done—eg analyses of subgroups and interactions, and sensitivity analyses	11
**Discussion**			
Key results	18	Summarise key results with reference to study objectives	12
**Limitations**			
Interpretation	20	Give a cautious overall interpretation of results considering objectives, limitations, multiplicity of analyses, results from similar studies, and other relevant evidence	12-14
Generalisability	21	Discuss the generalisability (external validity) of the study results	12
**Other information**			
Funding	22	Give the source of funding and the role of the funders for the present study and, if applicable, for the original study on which the present article is based	20

*Give information separately for cases and controls in case-control studies and, if applicable, for exposed and unexposed groups in cohort and cross-sectional studies.

Note: An Explanation and Elaboration article discusses each checklist item and gives methodological background and published examples of transparent reporting. The STROBE checklist is best used in conjunction with this article (freely available on the Web sites of PLoS Medicine at http://www.plosmedicine.org/, Annals of Internal Medicine at http://www.annals.org/, and Epidemiology at http://www.epidem.com/). Information on the STROBE Initiative is available at www.strobe-statement.org.

For peer review only
